# Longitudinal social contact data analysis: insights from 2 years of data collection in Belgium during the COVID-19 pandemic

**DOI:** 10.1186/s12889-023-16193-7

**Published:** 2023-07-06

**Authors:** Neilshan Loedy, Pietro Coletti, James Wambua, Lisa Hermans, Lander Willem, Christopher I. Jarvis, Kerry L. M. Wong, W. John Edmunds, Alexis Robert, Quentin J. Leclerc, Amy Gimma, Geert Molenberghs, Philippe Beutels, Christel Faes, Niel Hens

**Affiliations:** 1 Data Science Institute, I-BioStat, Hasselt University, Hasselt, Belgium; 2 Centre for Health Economics Research and Modelling Infectious Diseases, Vaccine & Infectious Disease Institute, University of Antwerp, Antwerp, Belgium; 3 Centre for Mathematical Modelling of Infectious Diseases, Department of Infectious Disease Epidemiology, Faculty of Epidemiology & Population Health, London School of Hygiene & Tropical Medicine, London, United Kingdom; 4 Department of Infectious Disease Epidemiology, Faculty of Epidemiology and Public Health, London School of Hygiene & Tropical Medicine, London, United Kingdom; 5 Epidemiology and Modelling of Bacterial Escape to Antimicrobials, Institut Pasteur, Paris, France; 6 School of Public Health and Community Medicine, The University of New South Wales, Sydney, Australia; 7 L-BioStat, Department of Public Health and Primary Care, Faculty of Medicine, KU Leuven, Leuven, Belgium

**Keywords:** Bias assessment, Social contact data, COVID-19, SARS-CoV-2, Survey fatigue, Under-reporting

## Abstract

**Background:**

During the COVID-19 pandemic, the CoMix study, a longitudinal behavioral survey, was designed to monitor social contacts and public awareness in multiple countries, including Belgium. As a longitudinal survey, it is vulnerable to participants’ “survey fatigue”, which may impact inferences.

**Methods:**

A negative binomial generalized additive model for location, scale, and shape (NBI GAMLSS) was adopted to estimate the number of contacts reported between age groups and to deal with under-reporting due to fatigue within the study. The dropout process was analyzed with first-order auto-regressive logistic regression to identify factors that influence dropout. Using the so-called next generation principle, we calculated the effect of under-reporting due to fatigue on estimating the reproduction number.

**Results:**

Fewer contacts were reported as people participated longer in the survey, which suggests under-reporting due to survey fatigue. Participant dropout is significantly affected by household size and age categories, but not significantly affected by the number of contacts reported in any of the two latest waves. This indicates covariate-dependent missing completely at random (MCAR) in the dropout pattern, when missing at random (MAR) is the alternative. However, we cannot rule out more complex mechanisms such as missing not at random (MNAR). Moreover, under-reporting due to fatigue is found to be consistent over time and implies a 15-30% reduction in both the number of contacts and the reproduction number ($R_0$) ratio between correcting and not correcting for under-reporting. Lastly, we found that correcting for fatigue did not change the pattern of relative incidence between age groups also when considering age-specific heterogeneity in susceptibility and infectivity.

**Conclusions:**

CoMix data highlights the variability of contact patterns across age groups and time, revealing the mechanisms governing the spread/transmission of COVID-19/airborne diseases in the population. Although such longitudinal contact surveys are prone to the under-reporting due to participant fatigue and drop-out, we showed that these factors can be identified and corrected using NBI GAMLSS. This information can be used to improve the design of similar, future surveys.

**Supplementary Information:**

The online version contains supplementary material available at 10.1186/s12889-023-16193-7.

## Introduction

Infectious respiratory diseases, in particular, are highly transmissible and a major cause of morbidity and mortality around the world [1]. Understanding the symptoms, causes, and transmission routes can help prevent and mitigate the resulting public health burden. Research in social networks has shown that the intensity of human-to-human interactions determines transmissibility and thus the effectiveness of many interventions [2]. Social contact information is essential in this context to understand the dynamics of transmission because these are driven by human behavior. Hence, data on close social contact in a population is a key component in understanding virus transmission dynamics, particularly respiratory viruses. Previous social contact studies have continually shown the importance of such data for parameterizing mathematical disease models, built to describe and understand the transmission dynamics of these infectious diseases [3]. The transmission parameters are strongly influenced by the integrated mixing patterns of a transmission model [4]. Several studies [5–7] have used social contact surveys to draw insights in how people mix in the population, which have then been used to guide the implementation of non-pharmaceutical interventions (NPI), such as in the context of COVID-19 [8–17].

As COVID-19 is primarily a close-contact transmitted disease, data on social contacts is crucial towards understanding the disease’s evolution. Several contact studies [10, 16, 18–21] have been conducted as the COVID-19 pandemic began to spread around the world to help understand the dynamics of the infections in the population, the impact of NPIs, and the optimal allocation of such measures [8]. The CoMix survey has been collecting multiple waves of a representative sample of data on social contact behavior since March 2020, in order to gain insights into behavioral changes. This longitudinal study gathers information on attitudes, awareness, and behavior in response to COVID-19 over time in Europe [16]. Nevertheless, as a long-term longitudinal survey, the CoMix survey could be subjected to fatigue effects that might appear when the survey’s participant becomes bored or less interested in the survey [22]. Such a respondent’s fatigue effect is a well-known phenomenon, leading to deteriorating data quality [23–25]. For example, participants may skip questions, spend minimal time answering questions (which will lead to increased variability and/or bias, e.g., because of under-reporting). Ultimately, survey fatigue may even prompt respondents to drop out of the survey entirely. As a result, survey response fatigue might be a potential threat to panel surveys and to surveys that use diaries to gather information [23, 26]; these effects can influence validity of the response and impact statistical analysis.

A first-order autoregressive logistic regression model was developed to examine how previous measurements impact current measurements, in order to enhance understanding of the dropout mechanism. This method was chosen for its simplicity and ease of interpretation, as it can be formulated as an independent-data GLM [27]. While previous research has focused on mitigating survey fatigue through study design [28, 29], our primary contribution lies in further addressing this issue at the data analysis stage. We adapted the approach of Backor et al. [26] to a social contact setting. Backor et al. described a similar way of correcting for under-reporting due to fatigue in a time-use study by calculating the number of activities a person would have reported in different time blocks throughout the day if this had been asked during the first hours of the survey. In the context of CoMix, under-reporting due to fatigue was accounted for by estimating fatigue effects and using the obtained estimates to predict the number of contacts that would have been observed when the survey had been taken for the first time. The Generalized Additive Model for Location, Scale, and Shape (GAMLSS) was used to model the number of reported contacts by taking into account this under-reporting. To the best of our knowledge, this is the first attempt to address under-reporting caused by response fatigue in a longitudinal social contact survey using this method. The approach we used to estimate the effect of under-reporting at various survey waves is applicable in any longitudinal survey context. Our findings may improve future analyses of the data that has already been collected, or used to assess bias in future surveys. Furthermore, we describe changes in social contact behavior from November 2020 to March 2022, both with and without correcting for under-reporting due to fatigue.

## Methodology

### CoMix study

CoMix is a longitudinal multi-country behavioral survey that was designed to monitor public awareness and behavior during the COVID-19 pandemic. Survey data were collected in the United Kingdom (UK), Belgium, and the Netherlands from March 2020 onward [16]. In Belgium [10], the sample was selected bi-weekly from existing panels of people who frequently partake in online surveys and was chosen to be representative of the Belgian population based on age, gender, and region of residence. Participants’ age, education level and occupation were recorded, together with information regarding their socio-economic status, health status, whether they experienced symptoms, and attitude towards and adherence to NPIs (limited to the first data collection period). In each wave, participants were asked to report all contacts made on a specific day between 5am on the day preceding the survey day and 5am of the survey day. A contact was defined as either an in-person conversation of three or more words in the physical presence of another person with (such as kiss or handshake) or without skin-to-skin contact [30]. Methodological details have been reported elsewhere [10, 12, 16].

The CoMix survey in Belgium spanned two data collection periods. The first period consisted of eight waves of data collection (April 2020 to August 2020) in a representative panel of adults (18 years and above) in terms of age, gender, and geographic area to have the same people participate in each wave. Children were omitted in the first period in order to get ethical clearance as quickly as possible. These 8 waves corresponded to different NPI regimes (Fig. S1). As a result of respondents dropping out in subsequent waves of data collection, the panel was supplemented with new members to meet the target quota.

**Fig. 1:**
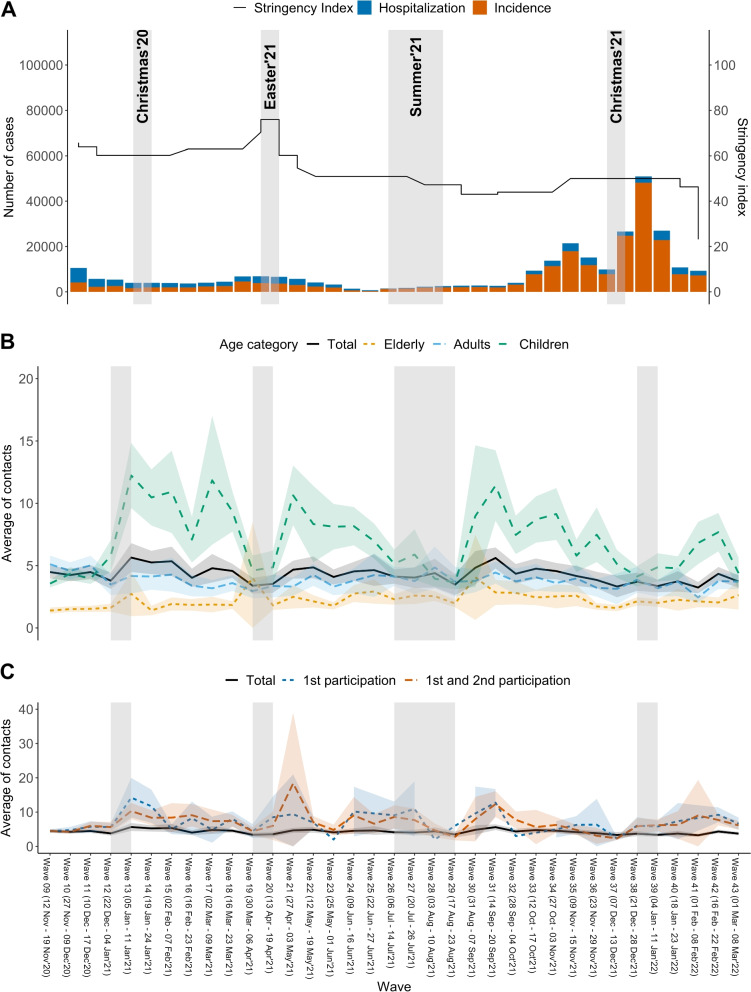
Calendar of non-pharmaceutical interventions (NPIs) and CoMix waves for the second survey period (waves 9-43) (1) From November 2, 2020, a face mask was required in any public place where a minimum 1.5-metre distance could not be guaranteed and was compulsory for everyone aged 12 and over in indoor and outdoor public spaces, catering industries, public transportation, and all organised public events. In October 2021; employees who work in Flanders did not have to wear a face mask at work anymore but masks were still required in stores, shopping malls, healthcare facilities, concert halls, sports centers, libraries, and places of worship as of October 29. On the other hand, masks are not required in locations where the Covid Safe Ticket (CST) is used. From March 2022, the use of face masks was no longer compulsory, but still recommended in the workplace. They were only required at that point on public transport,in hospitals and in residential care centres. (2) The Covid Safe Ticket (CST) had very limited usage in Belgium in July 2021 (only events > 1500 people) though it started to be used more widely as late as mid-October 2021. From early-mid November 2021, CST was needed throughout the country for visiting catering industry, theatres, concert halls, cultural centres, cinemas, museums, indoor amusement parks, public and private events for 50 people indoors and 100 people outdoors

**Fig. 2:**
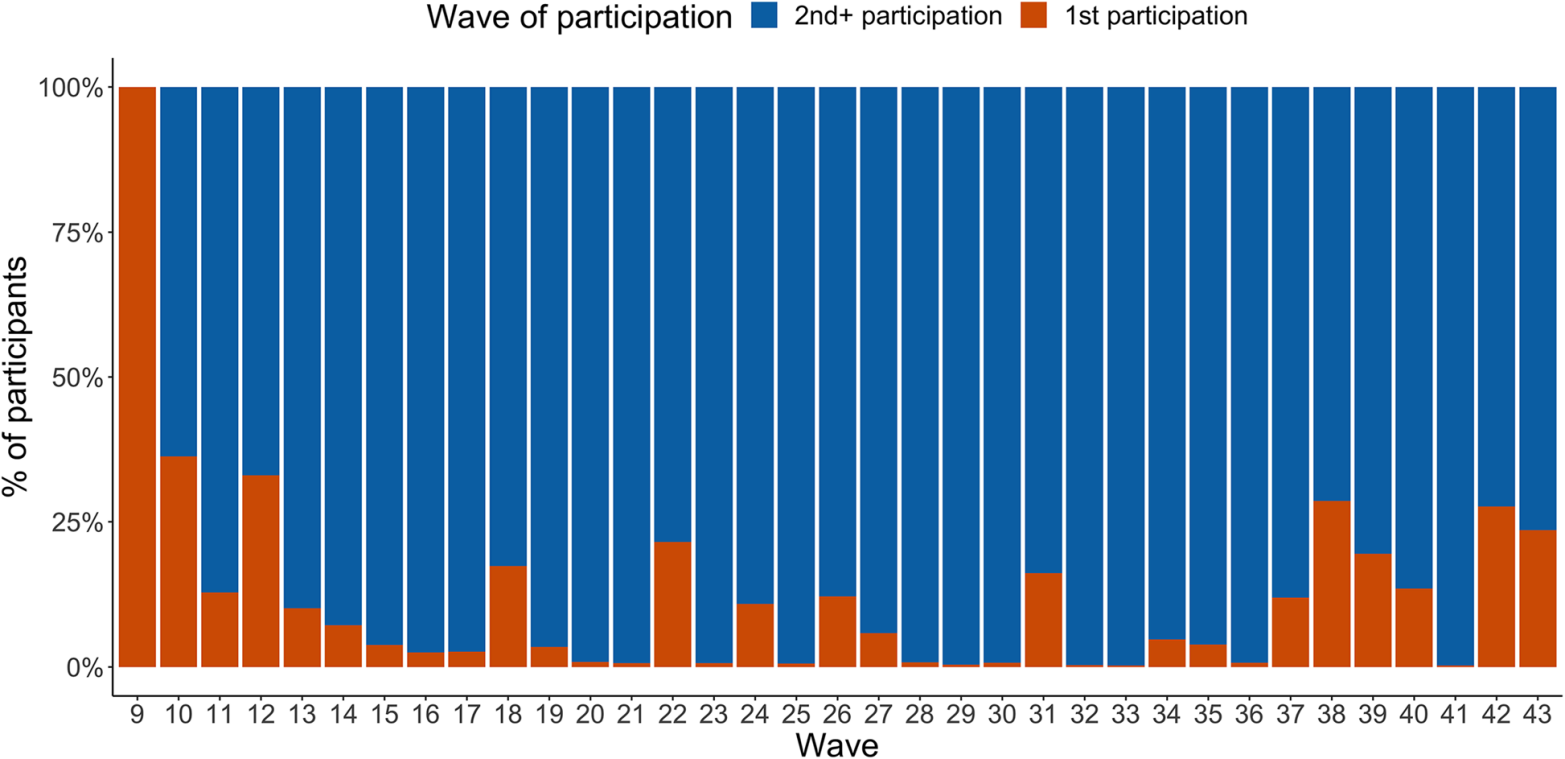
Participants proportion based on wave of participation in Belgium CoMix survey

The second survey period (November 2020 to March 2022) included 35 data collection waves (numbered from 9 to 43) and covers periods of both high and low SARS-COV-2 circulation and corresponding burden of disease in Belgium (for details, see Tables S1–S3). In the second period, a change in design was implemented to allow children’s data to amass. The questionnaires for children were completed by a parent within their household as a proxy. Given that the second period of the survey involved children, we focused on this part of the longitudinal survey (wave 9 - wave 43) to investigate how fatigue affects survey responses throughout the population. Therefore, we assume that wave 9 is the first wave of participation. Although participants may have taken part in the previous data collection, this ended 3 months earlier, and we assume that this lag was sufficient to eliminate possible fatigue bias from the previous data collection. Similarly, the cohort was continuously replenished to meet sample size requirements during subsequent waves of data collection due to participants dropping out (see Figs. 1, 2, and S2 for details).

### Degree distribution

#### Overall degree distribution

While generalized linear mixed models (GLMM) and generalized additive mixed models (GAMM) are constrained to the exponential family of distributions, GAMLSS are an extension of GAMM that allow for a broader range of conditional distributions of the response [31]. In GAMLSS, all four parameters (mean, variance, skewness, and kurtosis) can be modeled, either using linear parametric, non-linear parametric, or non-parametric (smooth) functions of the predictors, as well as normal or non-parametric random effects [32]. Negative Binomial [6] regression (R package *‘gamlss’* [31]) was used to model the reported number of contacts as a function of covariates and additive terms. However, as an extension of the GAMM model, GAMLSS does not provide parametric marginal regression functions by default, despite the fact that such functions are required for population-averaged inferences. Given that marginal and random-effects models have completely distinct interpretations [27], the marginal models are preferable when inferences about the population-average are the focus [33]. Marginal interpretation of negative binomial (NBI) [34] GAMLSS model can be derived by building a connection between the aforementioned model with the combined model for count data (See Additional file 2) [35, 36]. Thus, with appropriate precautions, all parameters in the NBI GAMLSS model, except the intercept, will have a marginal interpretation.

We defined 3 age groups (0–17 years (Children), 18–69 years (Adults), and 70 years and over (Elderly)) to build age-specific comparisons on the average reported contacts in a day. We selected three age groups because they reflect the various social contact patterns found in society. For each wave, the average number of contacts with and without correcting for the under-reporting due to fatigue were compared. Socio-demographic characteristics (household size [4] and area of residency), number of waves participation, participants’ health status and microscopic time settings (weekdays/weekends and regular/holiday period) [6, 37] were included as possible determinants, as well as face masks use. Here, the holiday periods refer to school holidays (e.g., Christmas, Easter, and Summer holidays), as well as a one-day national holiday in Belgium (e.g., Armistice Day). To exclude demographic inconsistencies, the household sizes are categorized based on the age categories (see Table S1 for detail on variables used in the analysis). Additionally, interactions between microscopic time settings [38], household size and area of residency, together with household size and holiday period were retained in the analyses. These covariates were chosen on the analysis to model the reported number of contacts. In addition, a random intercept for each participant and smoothing parameters (penalized varying coefficient [39] for Adults and Elderly, and cubic spline [40–42] for Children) on the survey days were used as additive terms (see Fig. S5 for detail). We considered socioeconomic status when constructing the model to identify factors influencing social contacts, but found that its inclusion did not substantially improve the model’s performance (see Table S4). As the models were already quite complex and considering that it was created based on three aggregated age categories, we expect that the remaining variables are more impactful. Additionally, information on vaccination and symptomatic status of Children was limited and as such not considered for analysis.

#### Mixing patterns in the degree of distributions

To attain insight into potential differences in mixing behavior between age groups, a NBI GAMLSS approach (as described in the previous section) was developed to estimate the age-specific average number of contacts for all combinations of contacts between age categories. For the purpose of inspecting the effect of mixing patterns, both age categories for participants and contacted individuals were taken into account. Socio-demographic (household size and area of residency), number of waves participated, the health status of participants, microscopic time settings, and face masks use were included as possible determinants. Moreover, smoothing parameters (penalized varying coefficient including interaction with participant symptomatic status) on the survey days, as well as random intercept for each participant was used as additive terms. Additionally, participant vaccination status was excluded from the model as it caused convergence problems.

Using social contact matrices created with the *‘socialmixr’* package [43], we compare age-group-specific social contact rates in the study period. This comparison can be used to examine the impact of under-reporting due to fatigue on estimating the reported number of contacts between age groups. The World Population Prospects of the United Nations [44] was used as the population reference, and weights were limited to a maximum of three to limit the influence of single participants, as used by Willem et al. [45]. To account for variability in the model prediction, contact matrices were produced based on 500 Negative Binomial simulations from the NBI GAMLSS model results (both with and without correction for under-reporting due to fatigue) for each predicted value of each participant. Then, we generated 500 bootstrap samples [46] with replacement of survey participants weighted by the age distribution of the actual population and weekdays and weekends to account for the uncertainty of the contact matrices and sample representativeness of the contact matrix [11, 21, 45]. The social contact matrix $\textbf{M}$ with elements $m_{ij}$ corresponding to the average contacts reported daily between each age group, which can be estimated by:

$$\begin{aligned} m_{ij} = \frac{\sum _{r=1}^{R_{i}} w^{d}_{ir} y_{ijr}}{\sum _{r=1}^{R_{i}}w^{d}_{ir}} \end{aligned}$$

where $w^{d}_{ir}$ represents the weight for participant *r* of age categories *i* who was surveyed on day $d \in \{\text {weekday, weekend}\}$ and $y_{ijr}$ defines the reported number of contacts made by participant *r* of age *i* with someone of age *j*. Naturally, contacts are reciprocal and thus $m_{ij}N_{i}$ should be equal to $m_{ji}N_{j}$. Reciprocity can be introduced to resolve reporting differences by:

$$\begin{aligned} m^{\text {reciprocal}}_{ij} = \frac{m_{ij}N_{i} + m_{ji}N_{j}}{2N_{i}} \end{aligned}$$

with $N_{i}$ and $N_{j}$ corresponding to the population size in age class *i* and *j*, respectively [30]. It is necessary to point out that the application of reciprocity is optional and should not always be imposed since this behavior might not be valid for specific contact types [45].

Furthermore, the per capita contact rate for participants of age *i* with individuals of age *j* in the population can be denoted by $c_{ij}$. A matrix $\textbf{C}$, called the contact rate matrix, is made up of the elements $c_{ij}$. This matrix is linked to the social contact matrix by $c_{ij} = \frac{m_{ij}}{N_{j}}$. In addition, percentages of change will be calculated by dividing the change in the number of contacts by the number of contacts before correcting for fatigue, then multiplying by 100.

#### Fatigue effect, missingness and dropout

Aside from under-reporting due to fatigue, it is possible that data are incomplete if respondents intentionally or unintentionally skip certain scheduled surveys or even drop out entirely. Drop out in a longitudinal study is defined as a participant terminating the study early due to circumstances beyond the investigator’s control [27]. In 1976, Rubin categorized incomplete data into three mechanisms: missing completely at random (MCAR), missing at random (MAR), and missing not at random (MNAR) [47]. In case when the missingness is independent of both unobserved and observed data, there are no systematic differences between observed data, perhaps conditional upon covariates, and missing measurements can be referred to as missing completely at random (MCAR). In contrast to MCAR, missing at random (MAR) occurs when the missingness depends on the observed data but not the unobserved data. Missing not at random (MNAR) mechanism indicates neither MAR nor MCAR, meaning the missingness depends on both unobserved data, even given observed data.

In this study, we define under-reporting as the discrepancy between the average number of reported contacts by respondents and the average number of contacts under the assumption that participants were taking the survey for the first time. Under-reporting due to fatigue was accounted for by estimating fatigue effects and using the obtained estimates to predict the number of contacts that would have been observed when the survey would have been taken for the first time. Here, it is assumed that intermittent missingness did not affect fatigue. Using GAMLSS, the average number of reported contacts can be modelled as

$$\begin{aligned} log(m_{itj}) = \beta _0 + \beta _{1}X_{1t} + \sum _{n=2}^{k} \beta _{nt}X_{nt} + \sum h_{tj}(x_{tj}) + u_{i} \end{aligned}$$

and the average number of reported contacts with fatigue correction can be obtained by

$$\begin{aligned} log(m_{itj}) = \beta _0 + \beta _{1} (1) + \sum _{n=2}^{k} \beta _{nt}X_{nt} + \sum h_{tj}(x_{tj}) + u_{i} \end{aligned}$$

with $m_{it}$ corresponding to the number of reported contacts of individual *i* measured in wave *t*. $\beta _1$ corresponds to the effect of participation to the first wave, and $\beta _n$ corresponds to other covariates used in the model. $h_{tj}$ is a smooth non-parametric function of the survey date *j*, while $u_i$ represents the random effects for each participant. By substituting the value of $X_{1t}$ with 1, we reflect participants are participating for the first time, hence correcting for under-reporting due to fatigue.

A first-order auto-regressive logistic regression model for the dropout process [27, 48] was developed by modelling the outcome as a function of previous outcomes for the same subject. This analysis was performed to see if current and previous values, as well as other covariates, had an effect on participants dropping out. A data set containing participants dropping out with dropout indicator as a response was constructed. The dropout indicator was assumed to follow a binomial distribution. Socio-demographic features such as gender, area of residency, and socio-economic status were used as predictors. By utilizing *‘gam’* package [49], random intercepts for each participant and cubic spline on the standardized days were used as smoothing parameters.

### Next generation principle

The next generation matrix $\textbf{G}$, in which the elements $g_{ij}$ correspond to the average number of secondary infections in age class *i* induced by the introduction of a single infectious individual from age class *j* into a fully susceptible population, can be used to model transmission dynamics [50]. The next generation matrix is defined by:

$$\begin{aligned} \textbf{G} = \frac{ND}{L} \varvec{\beta }, \end{aligned}$$

with population size *N*, average duration of infectiousness *D* and life expectancy *L* [6, 50]. The matrix of per capita rates $\beta _{ij}$ at which a person of age class *i* makes effective contact with a person of age class *j* is denoted by $\varvec{\beta }$. Based on the social contact hypothesis [3], it is assumed that individuals are contacted randomly within age class, with a proportionality factor *q* expressing specific disease infectivity and susceptibility and stipulating $\beta _{ij} = q \cdot c_{ij}$ [6]. Two assumptions on the proportionality factor (*q*) can be used in this case: homogeneous and heterogeneous proportionality factors. The homogeneous proportionality factor assumes that disease infectivity and susceptibility are both the same across age groups. The transition from a homogeneous proportionality factor *q* to a heterogeneous proportionality factor $q_{ij}$ is attained by assuming:

$$\begin{aligned} q_{ij} = \tilde{q} a_{i} h_{j}, \end{aligned}$$

with $a_{i}$ being a vector representing *q*-susceptibility, $h_{j}$ representing *q*-infectivity, and any residual effect is captured by $\tilde{q}$, as a remaining global proportionality factor. The computation of the relative incidence (*w*) is unaffected by the remaining factor. The vectors $a_{i}$ and $h_{j}$, however, only have a relative interpretation because of the presence of $\tilde{q}$. For the purpose of this study, we use $a_{i} = (0.39, 0.83, 0.74)$ and $h_{j} = (0.55, 0.79, 0.99)$ as the average $a_{i}$ and $h_{j}$ used in Franco et al. [51] for Children, Adults, and Elderly age categories, respectively.

The dominant eigenvalue of $\textbf{G}$ matrix can be denoted as $R_0$ [50], and is widely used to indicate whether an epidemic occurs ($R_0>$1) or dies out ($R_0 \le$1). To determine the relative change in $R_0$ from the one with ($R_{0,1}$) and without ($R_{0,2}$) correction for under-reporting due to fatigue, we calculate

$$\begin{aligned} \frac{R_{0,1}}{R_{0,2}} = \frac{\text {max}\left( \text {eigen}(\frac{ND}{L} q c_{1})\right) }{\text {max}\left( \text {eigen}(\frac{ND}{L} q c_{2})\right) }. \end{aligned}$$

In this case, it can easily be demonstrated that the normalizing constants cancel, and thus the ratio only applies to contact data [6]. Additionally, 95% percentile confidence intervals for the relative change in $R_{0}$ were calculated using a non-parametric bootstrap based on the aforementioned simulations.

Moreover, the ratio of the eigenvalues of two next generation matrices can be used to compare the relative temporal change in the reproduction number ($R_{t}$) using the next generation approach [51]. $R_{t}$ was used and calculated from the daily number of cases, and hospitalizations [52, 53] as a comparison by considering the time delays associated with the COVID-19 disease burden. When comparing $R_{t}$ estimates to the reproduction number calculated from the number of confirmed cases, a time shift of 7 (14) days is taken into account (respectively hospitalizations) [51, 54]. We set the reproduction number for CoMix wave 9 to equal the reproduction number calculated from infections or hospitalizations since the reproduction number is known up to the overall constant [51, 55]. Uncertainty due to sampling variability is estimated via non-parametric bootstrap.

Furthermore, the corresponding right and left-eigenvector of $\textbf{G}$ can be used to estimate the relative incidence *w* and the incidence rate *v*, respectively (see [51, 56] for details). Because the relative incidence *w* (usually normalized such that $\sum _{i}^{}w_{i} = 1$) is independent of disease-specific infectivity and susceptibility, it can be calculated directly from the social contact data. Nevertheless, since the eigenvector *w* can only be recovered up to a global constant, the age-specific component of the eigenvector has no meaning. Relative ratios such as $\frac{w_{i}}{w_{j}}$ can be interpreted nonetheless, providing an estimate of the relative incidence in age class *i* as compared to the incidence within age class *j*.

### The impact of interventions on mixing patterns

Both pharmaceutical (PI) and non-Pharmaceutical interventions (NPI) must be taken into account to frame and translate contact behavior into transmission dynamics. The GAMLSS model takes into account the use of face masks as one of the NPIs as well as vaccination status as a PI to the number of contacts reported in the survey. In addition, school and workplace closures, as well as travel restrictions, had an impact on mixing patterns [37, 57]. Here, we investigate these effects by comparing the average number of contacts in each survey wave and by investigating the relation between the stringency of NPIs in Belgium and the number of contacts reported in the CoMix survey.

**Fig. 3:**
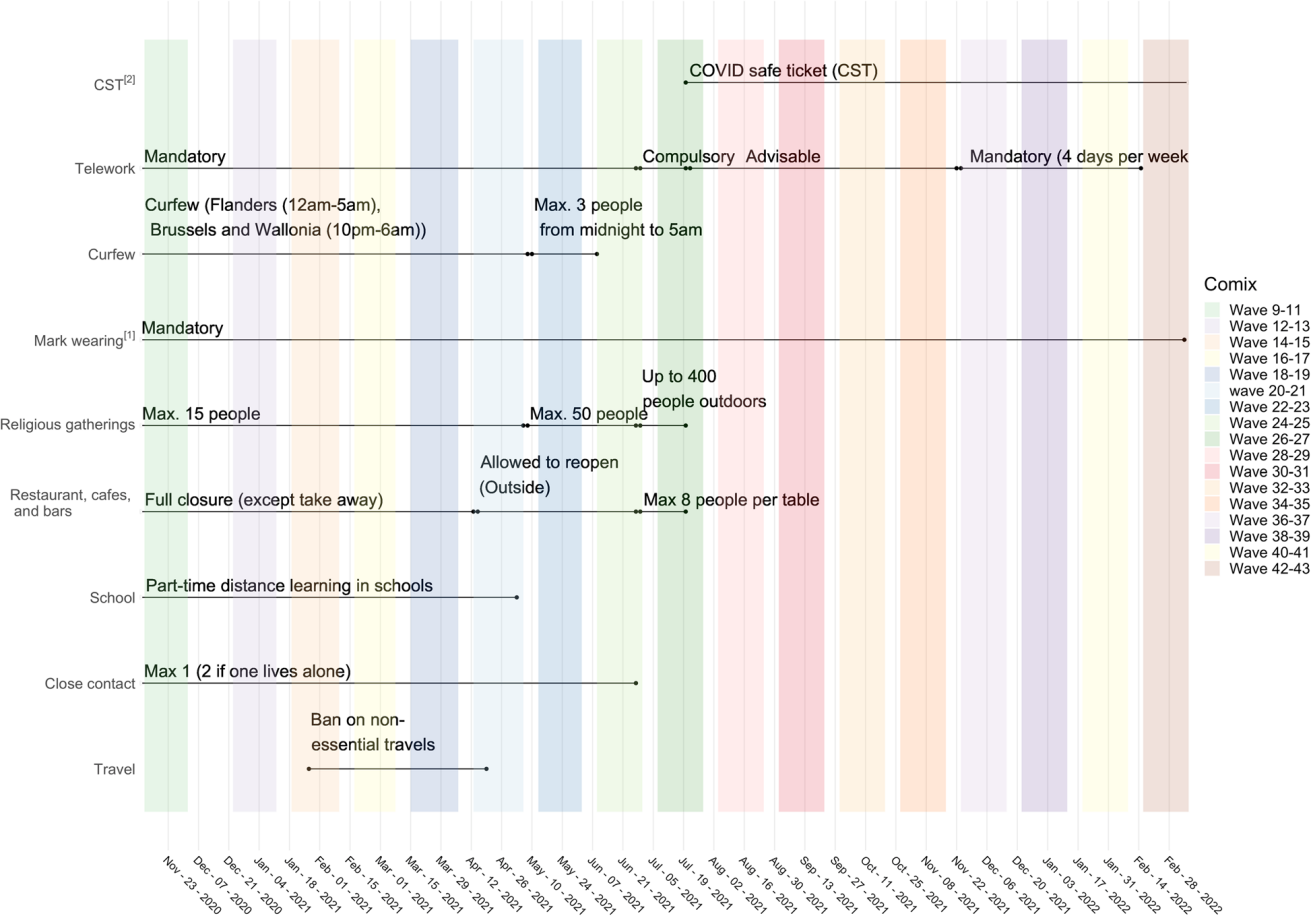
Average contacts over time by age categories and participation status with 95% CI. **A** Number of cases and hospitalisations due to COVID-19 in Belgium. **B** Average number of contacts and 95% CIs in Belgium for all participants, elderly only (>70 years), adults only (18–69 years), and children only (<18 years). **C** Average number of contacts and 95% CI in Belgium for different waves for all participants (participating from 1 up to 34 times), participants who participated for the first time, and participants who participated for the first and second time

The stringency index (Government Response Stringency Index) is a composite measure summarizing nine response instruments, such as school closures, workplace closures, and travel restrictions, re-scaled to a value of 0 (no interventions) to 100 (strictest response; Oxford COVID-19 Government Response Tracker, Blavatnik School of Government) (see [58] for a full description). Stringency index has been proven to correspond with a reduction of contacts in the community [8]. Here, we present the results for 35 waves collected with different stringency index values throughout the survey waves (see Fig. 3). To compare the number of contacts with and without correction for under-reporting resulting from fatigue, contact matrices representing mixing patterns throughout the study period were also estimated. The social contact matrices and their percentages of change were calculated in the same way as described in the section on mixing patterns, with the exception that each survey wave was calculated separately.

## Results

### CoMix study

A total of 4,592 (47% males and 53% females) participants reported 42,922 responses over 34 waves. The total number of reported contacts was 182,986 with the highest number of contacts reported by one participant being 744. The average participant age was 36 years, and people under the age of 18 made up for 30.4% of the sample, while the Elderly (i.e. above 70) represented 6.9%. The average household size was 3, ranging from 1 to 12, with 90% of the participants living in a household of sizes smaller than 4. More than half of the survey participants come from the Flemish Region (55.4%), whereas 9.1% come from the Brussels Capital Region, in agreement with the regions’ population sizes. The participants reported an average of 1.17 contacts per day with the median number of reported contacts being 2 (inter-quantile range (IQR):[1;4]).

Figure 3 depicts the number of cases and hospitalisations due to COVID-19 in Belgium and the stringency index, as well as the average number of contacts reported by participants in each wave, from wave 9 (12 November 2020) to wave 43 (08 March 2022). The gray shades represent the holiday seasons of Christmas 2020, Easter 2021, Summer 2021, and Christmas 2021, respectively. Figure 3A depicts the number of COVID-19 cases and hospitalizations in Belgium. During waves 19 and 20, there is a decline in the number of contacts reported by children. This could be attributed to the Easter pause that was implemented and an overall increase in the stringency index during this period [58, 59].

The age-specific contacts reported during the survey period are depicted in Fig. 3B, which shows the average number of contacts based on the CoMix survey for all participants and for the age groups of Elderly (70 years and over), Adults (18–69 years), and Children (0–17 years). It can be seen that Children (7.05 [8.83;5.26]), on average, have more contacts than Adults (3.81 [3.02;1.49]) and the Elderly (2.25 [3.02;1.49]). Adults, in comparison to Children and the Elderly, have less variability in the average number of contacts over time. It is worth noting that the average number of children’s contacts decreased significantly during both the Easter and Summer vacations. In Fig. 3C we also show the average number of contacts for participants reporting for the first time and for the first two times. It can be seen that people who participated for the first time (6.95 [3.81;11.3]) and the first two times (6.93 [3.79;10.2]) reported more contacts than the grand average (4.28 [3.50;5.06]). Note also that the total average of contacts of respondents participating from 1 up to 34 times exhibits less variability.

### Degree distribution

**Fig. 4:**
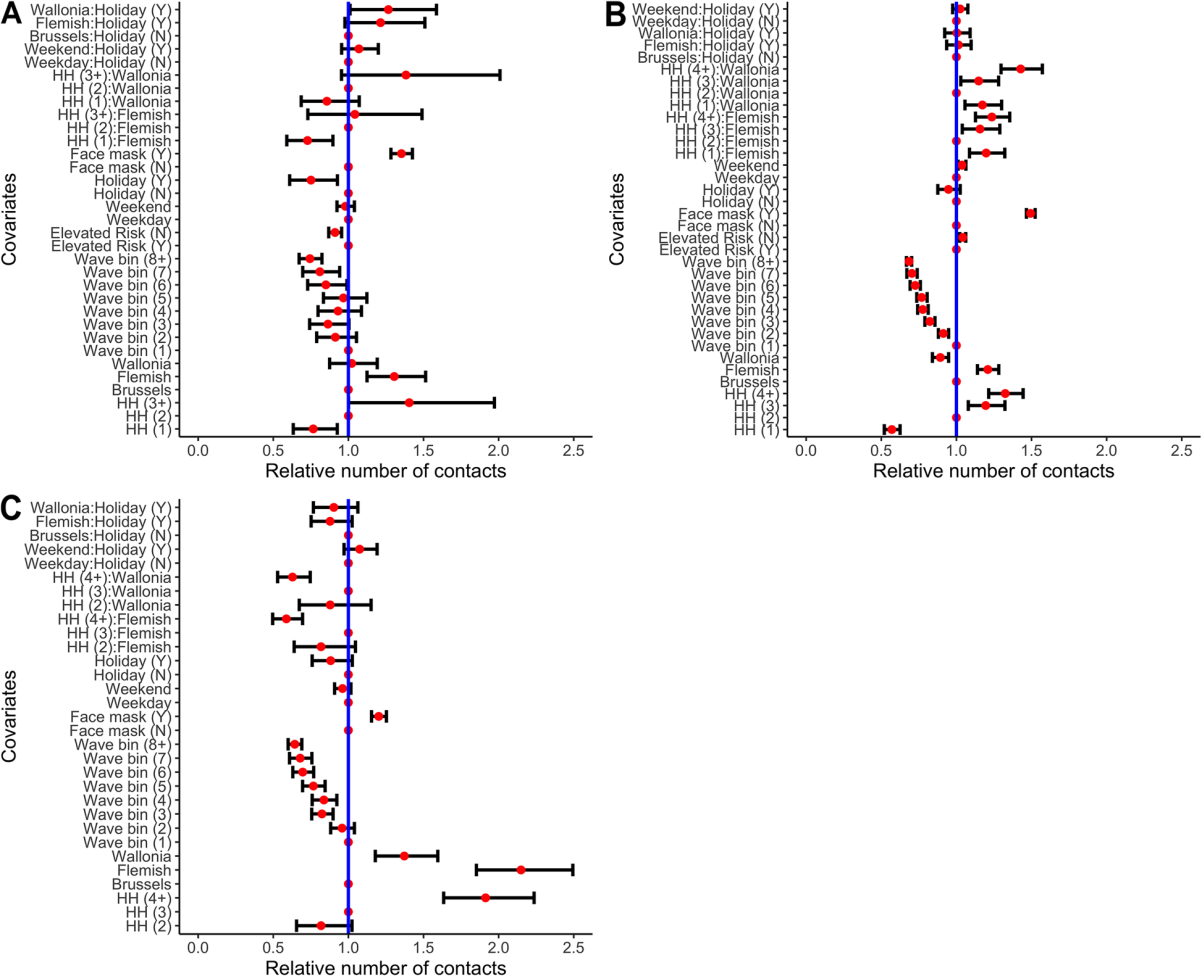
Relative number of contacts *(red dot)* and 95% CI based on NBI GAMLSS model; **A** *Elderly*, **B** *Adults*, and **C** *Children*

Figure 4 shows the summary results of the NBI GAMLSS model for the average number of contacts in different age groups (see Fig. S5 for model performance). The number of contacts is consistently influenced by household size and the use of a face mask. It is noted that participants who live in larger households have a higher number of contacts. In addition, the usage of face masks also consistently has a positive association with an increase of 35.3%, 49.4%, 20.2% for the Elderly, Adults, and Children, respectively. Elderly who are not in the high-risk category, reported fewer contacts (91.1%; CI = [86.9% ;95.5%]). Adults who are not in the high-risk category, on the other hand, reported more contacts (1.04 times higher). This information, however, was not collected for the children in the survey. Additionally, microscopic time settings (holiday/non-holiday or weekend/weekday period) do not significantly affect the number of contacts reported with Adults and Children. Holidays, on the other hand, show a negative correlation with the number of contacts reported among Elderly (75.1%; CI = [60.9%; 92.7%]), whereas weekend has no significant impact on the number of contacts reported. Equally importantly, there is a clear downward trend in the number of contacts related to the number of waves people participate in. These declining patterns appear across all age categories; nevertheless, the effect is more noticeable in Adults and Children than in the Elderly.

#### Under-reporting due to fatigue

**Fig. 5:**
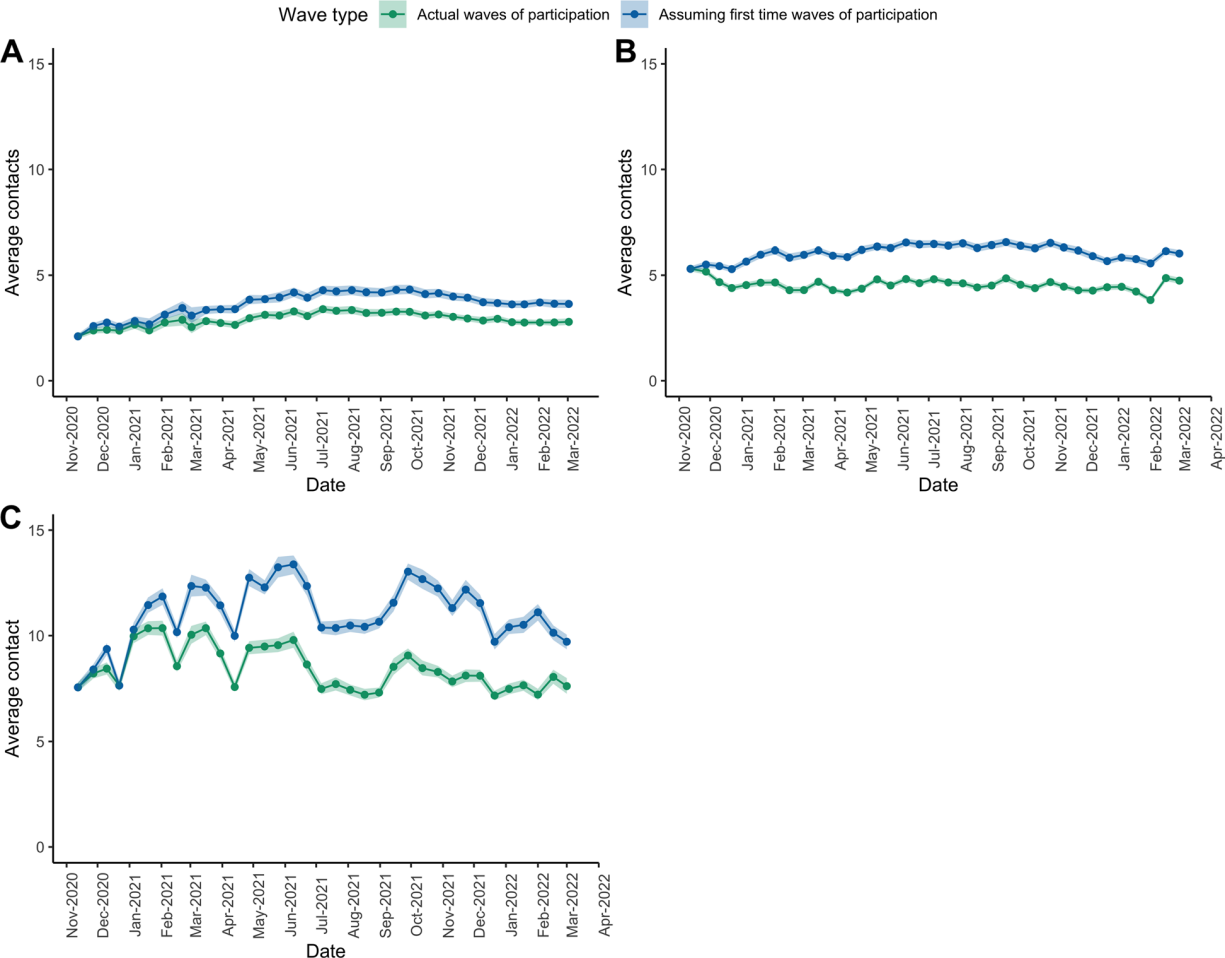
Expected average number of contacts. Both uncorrected *(green)* and corrected for fatigue effect *(blue)* based on NBI GAMLSS model; **A** *Elderly*, **B** *Adults*, and **C** *Children* with 95% Confidence Interval based on Non-parametric bootstrap

Figure 5 shows the difference in the marginal average of contacts across age groups with and without correction for under-reporting due to fatigue. It is noticeable that correction will consistently result in higher average number of contacts across all age groups.

#### Missingness and dropout

Out of 4,592 participants, 2,155 participants (46.92%) dropped out of the survey; out of these, 1,641 respondents (76%) only participated once in the survey (including new participants in Wave 43). Table 1 presents the results of the first-order auto-regressive logistic regression model for the dropout process. The effects ‘$Y_{i,j}$’ and ‘Dep. on $Y_{i,j-1} (1)$’ capture the current measurement and transition effect for the later measurement, respectively. These parameters have non-significant results, implying that the dropout is not dependent on the latest and its penultimate number of contacts reported. Given the model framework, this result shows that missingness under MCAR applies under the assumption that MAR is the alternative. Recall that ruling out MNAR is virtually impossible based on the observed data alone.

**Table 1:** Parameter estimates of the first-order auto-regressive logistic regression model showing the relative incidence (RI), the corresponding confidence intervals, and *p*-values for participant dropout. Categories with no values (“-”) are the reference categories

Estimate	RI	RI CI
Household size (1)	1.016*	(0.056,1.976)
Household size (2)	-	-
Household size (3)	-0.514	(-1.435,0.407)
Household size (4+)	-0.682	(-1.609,0.245)
Adult category (Children)	-	-
Adult category (Adult)	1.715*	(0.690,2.740)
Adult category (Elderly)	2.141*	(0.644,3.638)
Gender (F)	-	-
Gender (M)	0.305	(-0.318,0.928)
Area (Brussels Central Region)	-	-
Area (Flemish Region)	0.056	(-0.948,1.060)
Area (Wallonia Region)	-0.320	(-1.363,0.723)
Participant’s social group (1 &2)	-	-
Participant’s social group (3 &4)	0.222	(-0.652,1.096)
Participant’s social group (5 &6)	-0.052	(-0.989,0.885)
Participant’s social group (7 &8)	-0.046	(-0.959,0.867)
$Y_{i,j}$	-0.003	(-0.017,0.011)
Dep. on $Y_{i,j-1} (1)$	-0.011	(-0.025,0.003)

(*)*p*-value <0.05

### Mixing patterns

**Fig. 6:**
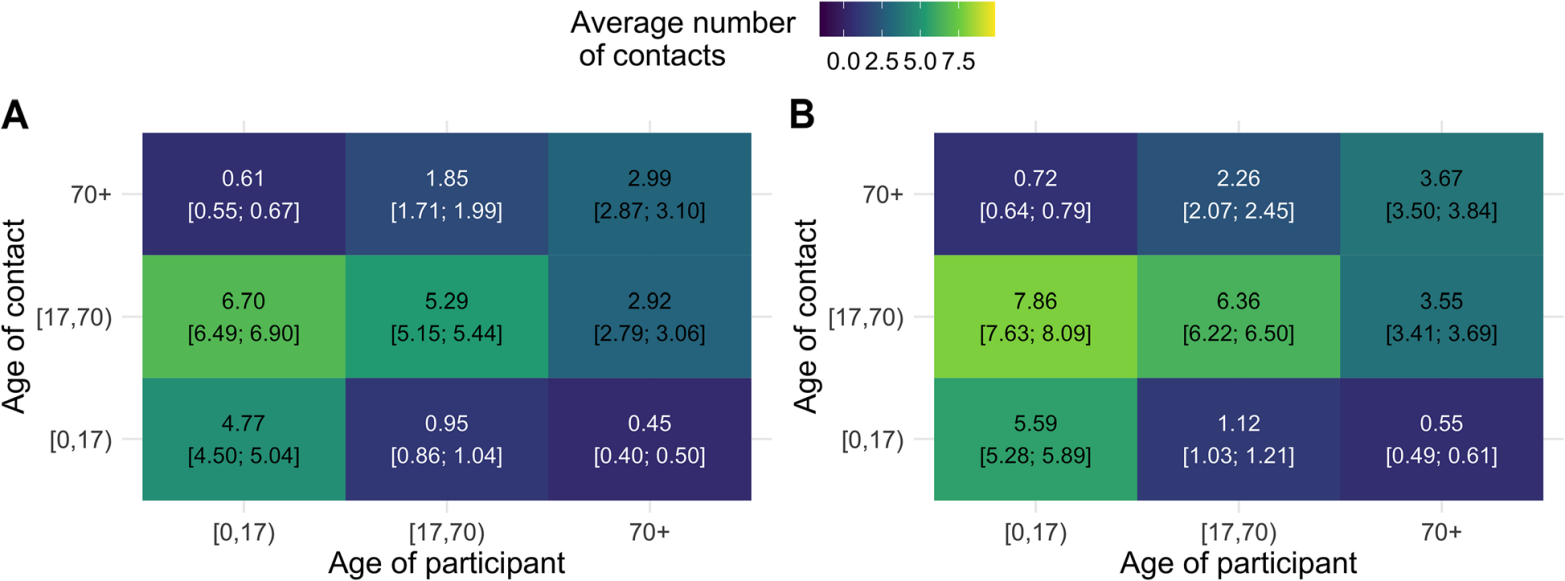
Social contact matrices; average number (wave 9–wave 43) of daily reported contacts; **A** *reported numbers;* **B** *corrected for under-reporting due to fatigue*

**Fig. 7:**
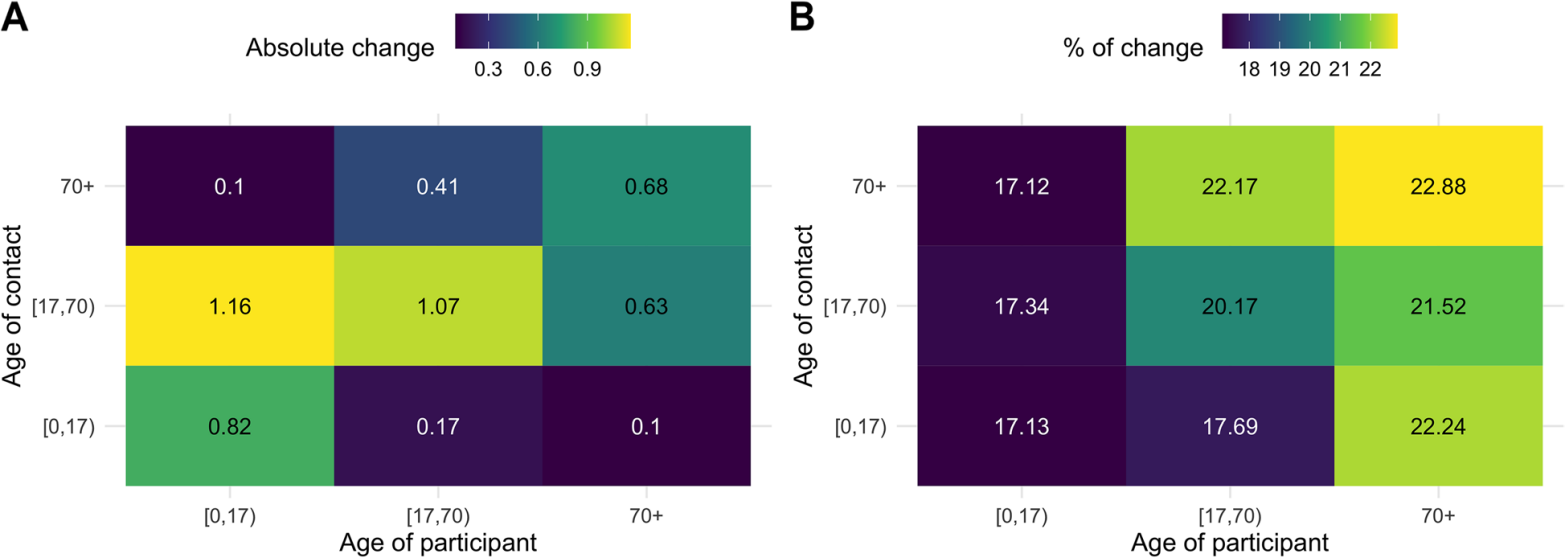
Changes between average number of contacts. Absolute change (**A**) and relative change (**B**) between average number of contacts with and without correction for under-reporting due to fatigue

Figure 6 depicts the social contact matrices (waves 9–43) weighted for the age and weekdays/weekends categories with and without correcting for under-reporting due to fatigue. It can be observed that the average number of reported contacts is considerably lower without correction *(left)* than with correction *(right)*. For participants–contacted (*without correction; correction* [95% CI]) age groups, most contacts occur between Children–Adults (6.70 [6.49; 6.90]; 7.86 [7.69; 8.09]), Adults–Adults (5.29 [5.15; 5.44]; 6.36 [6.22; 6.50]), and Children–Children (4.77 [4.50; 5.04]; 5.59 [5.28; 5.89]). The least interaction prevails between Elderly–Children (0.45 [0.40; 0.50]; 0.55 [0.49; 0.61]). In addition, Fig. S10 shows the average number of contacts when reciprocity is taken into account, in which the largest difference occurs between Children and Adult contacts. Additionally, Fig. 7 depicts the percentages of change between the average number of contacts with and without correction for under-reporting due to fatigue. Note that correcting this in the survey will increase the average number of reported contacts by $\sim$17% to $\sim$23%. On average, Children (0.66; 17.9%) receive the highest absolute change of correcting the number of reported contacts, while the Elderly (0.47; 22.21%) have the smallest. Furthermore, Children reporting contacts made with Adults receive the largest absolute change of correction (1.16; 17.34%), whereas Children and the Elderly contacts receive the smallest.

### The impact of interventions on mixing patterns

Asymmetric and symmetric wave specific social contact matrices are calculated with and without correction for under-reporting due to fatigue; they are displayed in Figs. S9 and S10. For each wave, in general, the highest average number of contacts occurs between Children (as survey participant) and Adults (as the person in contact), whereas Children (as survey participant) and Elderly (as the person in contact) show the least average number of contacts. The percentages of change between the number of contacts reported with and without correcting for under-reporting due to fatigue is depicted in Fig. S11. Since all participants participate for the first time on the 9-th wave, by definition, the percentages of change in the reported contacts are set to zero. As the number of survey waves increases, so does the percentages of change in the reported contacts. After Wave 19, the percentages of change begin to stabilize at around 25%-35%. It is also worth noting that as the number of new survey participants increases (Fig. 2), the average percentages of change decrease. As an illustration, in waves 38 and 42, there are more than 25% new participants, resulting in a decrease in the percentages of change in the contact matrices; whereas in waves 36 and 41, there is a high percentage of change in the number of contacts reported, due to a low number of new participants in the survey (less than 5%).

**Fig. 8:**
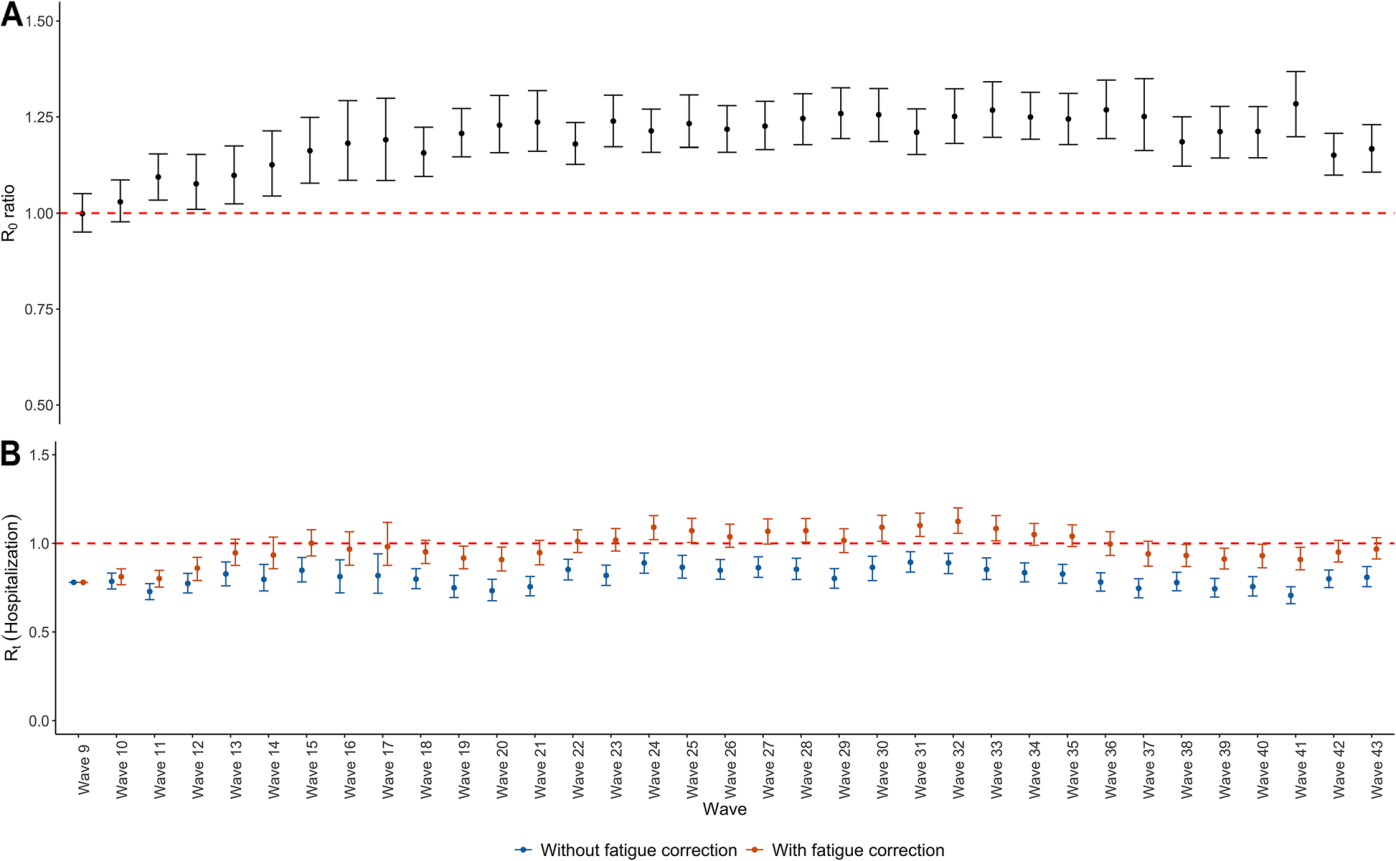
Effects on correcting for under-reporting due to fatigue in $R_0$. **A** Relative changes in $R_0$ with and without correcting for under-reporting due to fatigue and **B** The impact of correcting for under-reporting due to fatigue on the hospitalizations reproduction number estimated from heterogeneous CoMix data

The relative changes in $R_0$, comparing the effect of fatigue correction, are depicted in Fig. 8A, together with their 95% bootstrap-based confidence intervals. Omitting the fatigue correction had a moderate impact on $R_0$, as shown by the fact that it affected around $\sim$20% of the $R_{0}$ ratios, on average across the survey periods. This effect grows during the first few survey waves, after which it stabilizes until the end of the study period. Furthermore, Fig. 8 (Fig. S14) depicts the effect of correcting under-reporting due to fatigue on hospitalizations (COVID-19 incidence rate) reproduction number estimated from heterogeneous CoMix data, using the reproduction number from Wave 9 as a baseline. In addition, comparisons of the relative incidences using homogeneous and heterogeneous susceptibility and infectivity between age categories are depicted in Fig. S15. The relative incidence without and with correction for the under-reporting due to fatigue are represented in red and blue, respectively, with homogeneous and heterogeneous susceptibility and infectivity represented as a circle and triangle, respectively. It can be seen that there is a tiny difference in relative incidence with and without correction for both heterogeneous and homogeneous assumptions. Moreover, for each observed wave, the relative incidence among Adults is always the highest. Furthermore, it is clear that relaxing the homogeneity assumption, results in a change in the proportion of relative incidence.

## Discussion

In this study, the effect of fatigue in the second part of the CoMix survey in Belgium (12 November 2020 – 08 March 2022) was investigated. We examined the factors that influence the number of contacts reported as well as the mixing patterns for three age categories (Children, Adults, and Elderly). Missingness in the survey was also investigated. Note that, rather than considering the influence of survey fatigue on non-response and missing data, we focused on the impact of survey fatigue on the observable response. Furthermore, the impact of lockdown was estimated using the social contact matrix and the next generation matrix, both with and without correcting for under-reporting due to fatigue.

The study indicates that age was associated with number of contacts. Children reported the highest average number of contacts, a finding that was also found in the UK survey [9]. Household size has a coherent impact on the number of contacts reported, with a larger household size being associated with a higher number of contacts reported. This finding is in line with those of other studies [38, 60–62]; they all emphasize the role of household structure as a crucial driver for close-contact interactions. Area of residency also has a significant effect on the number of contacts reported, except for the Elderly living in Wallonia. People in Flanders are more likely to make contacts than those in Brussels and Wallonia, which is consistent with the first period of data collection (waves 1-8) [10]. Furthermore, there is some association with Adults who were not in an elevated risk group reporting higher contact rates. This effect, however, is reversed in the Elderly. The fact that the majority of Elderly in high-risk groups live in nursing homes may explain this finding since they have more opportunities to interact with others. It was also uncovered that people who wore face masks reported more contacts. Several phenomena could explain this rise. First, this may represent an increase in contacts outside the households, during which wearing a face mask may be either mandatory, or more likely if the participants perceive these contacts as higher risk and hence make a greater effort to guarantee safety. Second, people making more contacts may understand the risk of infection of this behavior and therefore are more likely to wear masks [61].

In addition, microscopic time (weekday/weekend or regular day/holiday) does not significantly affect the number of contacts reported for Children, although visually holidays seemed to be the main determinant of children’s contacts (3B). Holiday periods and their interaction for Children only show a barely significant association with a lower reported number of contacts. The Elderly reported less contact during holidays, while Adults reported more contact during weekends. In contrast to Hoang et al. [38] we did not find significant differences in the number of contacts reported for the interactions in microscopic time. This disparity might be explained by the study’s use of larger aggregated age classes, incurring some loss of information. Likewise, it could also be caused by a different way of people making contacts, as there is less of a discrepancy between holiday periods, weekdays and weekends in the pandemic setting. Moreover, there are no statistically significant differences in the number of reported contacts between the Elderly and Adults based on symptomatic and vaccination status. Although these effects are not significant, these parameters could be confounders as both of them could influence the mixing patterns. We also observed a high inter-generational age-mixing between adults and children, with most contacts reported in the Children-Adults bracket. This aligns with previous studies and can be explained by contact networks of families and working-age groups. Contact between elderly and children was the least reported, which is consistent with pre-pandemic findings [38]. These affirm that the reported number of contacts is impacted by the disparity in age distributions in the population. Finally, our findings highlight that as people participate for a longer time period in the survey, they tend to report fewer contacts; this effect is less pronounced in the Elderly. This phenomenon is likely attributable to survey fatigue, a common phenomenon in the collection of longitudinal survey data [63].

Given that dropouts were observed in the model, several baseline variables were used as covariates to investigate this pattern. A first-order auto-regressive logistic regression model was developed to investigate whether the current measurement of the number of contacts has any correlation with the previous measurement. There is no significant relationship between the dropout pattern and the current and previous number of contacts reported, as shown in Table 1. This is evidence for the fulfillment of MCAR assumption given that MAR is the alternative. Furthermore, participants living alone in a household, as well as the age categories have a significant influence on the drop out pattern, indicating a covariate-dependent MCAR. This is a subset of MCAR in which dropout is linked to observed covariates measured prior to dropout [64]. Given this assumption, one can analyze the data using a frequentist, likelihood, or Bayesian procedure while ignoring the process(es) of generating the missing values [27, 65]. However, we must re-emphasize that an MNAR process cannot be ruled out. We consider a sensitivity analysis to this effect outside of the scope of the current paper.

One of the most important findings of our study is that, despite the fact that different interventions were used throughout the study period, the fatigue effect across ages is remarkably consistent over time ($\sim$20%) after weighting the age and weekdays/weekends categories, with higher correction in survey waves with a larger number of new participants. This value applies to both the contact in the contact matrices and the $R_0$ ratio between correcting and not correcting for under-reporting due to fatigue, in which the effect can be seen on the reproduction number of hospitalizations and the number of incidence rates. The result shows that using the uncorrected contact data would lead to underestimated reproduction numbers. Furthermore, we also show that under-reporting can be estimated using a constant number (instead of as a function of time, for example, which might lead to a severe and more complicated effect). Additionally, we acknowledge that the 20% under-reporting due to fatigue is a real issue. As a result, we demonstrated how this effect is on the COVID-19 situation, where the effect of 20% is less severe because the under-reporting factor was multiplied by a small value of $R_0$. This effect will unarguably have a greater impact as the value of $R_0$ increases. Furthermore, as shown in Fig. 8 (Fig. S14), the relative changes in $R_0$ increase in the first few waves and then stabilize until the end of the study period. Additionally, it was found that correction for under-reporting due to fatigue did not change the pattern of relative incidence between age groups, when both homogeneous and heterogeneous infectivity across age groups were imposed (Fig. S15). These findings imply that under-reporting in the CoMix survey can be relatively well understood and corrected, provided some reasonable assumptions are made in the analysis.

Differing from the findings of the previous studies [10, 66], which concluded that lifting lockdowns in various periods in 2020-2021 resulted in increased social contact, the data from this Belgian contact survey did not demonstrate an apparent increase in the average number of contacts as interventions were relaxed between May 2021 and June 2021, despite the rise in observed mobility (Fig. S16) [67]. Although previous work have suggested a strong correlation between Google mobility data and CoMix data in an earlier phase of the pandemic [68], the use of Google data as a proxy for social contact data requires further investigation. One reason is that individuals, although resuming their usual mobility pattern to attend work and other activities, may still attempt to maintain physical distance when interacting with others. Additionally, it is essential to consider disparities in individuals’ mobile phone usage patterns, which can introduce significant biases. For example, variations may arise in how individuals of different age groups use their phones, potentially influencing the validity and reliability of mobile phone metrics within these specific populations [69].

The difference in the findings can be attributed to several factors. Firstly, participants awareness of the study focus on COVID-19 led to a sample that demonstrated heightened concern regarding the risks associated with the coronavirus, as shown by Kenedy et al. [70]. Considering the novelty of COVID-19, participants may have initially displayed a high level of motivation to contribute to the survey, thereby minimizing the presence of selection bias. However, it is important to acknowledge that as time progresses, participants’ perceptions of the pandemic severity may undergo changes, which could potentially introduce an elevated risk of selection bias in online surveys. While our sampling design aims to ensure representativeness regarding age, gender, and region of residence, it is important to acknowledge that there may be potential influence from other factors [61, 71], which could impede the ability to draw accurate conclusions [72, 73]. Secondly, Coletti et al. (2020) conducted a study that revealed a highly stringent lockdown measure with a stringency index above 80 in the first wave of the study. This included mandatory school and work-from-home policies, the closure of non-essential shops, and restrictions on non-essential travel outside Belgium [74]. Subsequently, there was a period of moderate stringency from early June to the end of July 2020, with the stringency index hovering around 50. During this time, there were partial reopenings of schools and workplaces, and shops were allowed to operate at maximum capacity [75]. Here, fluctuations in the stringency index can lead to significant variations in contact patterns. In April 2021, there was a relatively brief increase in stringency during the Easter pause. The stringency index rose to 76, accompanied by mandatory working from home, permission for non-essential shopping by appointment, a curfew, restrictions on social gatherings, and closure of non-medical professions. Subsequently, in May 2021, the stringency level decreased to 50. Non-medical contact professions were allowed to operate, curfews were replaced with restrictions on gathering sizes, and outdoor bars and restaurants were permitted to reopen [76]. Considering the mild change in the stringency index and in conjunction with a mild version of the aforementioned bias, it is plausible that the CoMix study did not reveal a substantial change.

The CoMix survey has some limitations, including the possibility of bias due to social desirability in self-completed surveys during the pandemic. This is based on the fact that such surveys specifically attract participants who adhere to social distancing rules. Participants may provide responses that they believe are socially desirable rather than reflecting their true behaviors, which can lead to an inaccurate representation of reality and affect the validity of study findings [77–79]. We sought to reduce this bias by setting up CoMix with an established online panel that did not focus on healthcare-related questions [12], and by ensuring participant anonymity. The retrospective nature of reporting social contacts may introduce another form of bias [9, 16, 70]. The accuracy of retrospective data, such as the number of contacts reported, may be compromised as participants face challenges in accurately recalling past events, resulting in inaccurate recollections and reduced reliability. Nevertheless, we believe the ramifications are limited since participants only reported contacts made within a recent time frame [10]. While this study successfully describes contact patterns for age categories throughout the study periods, these three age classes do not allow to fully capture the heterogeneity in the population contacts. However, including more age classes would result in data sparsity in some age classes. Furthermore, vaccination status was excluded from the mixing pattern analysis because it causes a convergence problem, despite the fact that it may be an important confounder. This convergence problem might be explained by constant values recorded in the vaccination status, though a deeper investigation is needed. Additionally, a more in-depth analysis of the missing data mechanism could be undertaken. A more complex method based on multiple imputation methods, for example, can be developed to adjust the analysis for the occurrence of non-response in several ways. However, doing so with the proposed model is computationally expensive. Lastly, we are aware that the CoMix data contains many individuals who only participate in the survey once and subsequently drop out, which we refer to as singletons [80]. Several studies have shown that small cluster sizes, when the number of subjects within the units is small, leads to biased estimates of both residual and random-effects variances in the linear mixed model [81]. Fortunately, both fixed-effects estimates and their standard errors were unbiased in the presence of small clusters [82, 83]. Here, we evaluate the significance of the presence of random-intercepts in the model using the Best Linear Unbiased Predictors Based Permutation Test [84] as an extension of the permutation test. The permutation-based test was used since it offers a more reliable alternative than the *F* test when many singletons are present [81]. Despite the presence of singletons, the test demonstrates a significant effect on the random intercept (result not shown). However, it should be noted that the majority of these studies were conducted in the linear-mixed model context, whereas this study was conducted in the GAMLSS context.

## Conclusion

This study highlights the importance of acknowledging the presence of under-reporting due to fatigue in longitudinal contact surveys in Belgium, which provided valuable information on human behavior in the transmission of airborne diseases. As the CoMix study can describe how people mix in the population, during the second data collection period, we discovered that there are, on average 20% under-reported contacts due to survey fatigue. This effect is more pronounced in waves with a small number of new participants than in waves with many new participants and vice versa. However, the effect is consistent throughout the study and can be well understood. Therefore, upon making reasonable assumptions in future analyses, the survey fatigue effect can be managed and will not unduly affect the epidemiological conclusions drawn from the survey.

## Supplementary Information

**Additional file 1.** Supporting information. This additional file provides supporting information to support the findings on the main article.

**Additional file 2.** Connection between GAMLSS for Counts and the Combined Model. This additional file provides the relationship between GAMLSS for counts and the combined model. The derivations depicted the connection between marginal and hierarchical interpretation of Negative Binomial I GAMLSS model.

**Additional file 3.** Ethical forms (in French and Dutch). This additional file provides the ethical forms of the survey, both in French and Dutch.

**Additional file 4.** CoMix study questionnaires in English. This additional file provides the CoMix study questionnaires in English.

## Data Availability

The datasets generated and/or analysed during the current study are available in the Zenodo-based repository [85], https://zenodo.org/record/7086043#.Y0Zt9C8RrmE, www.socialcontactdata.org/data, as well as on the CoMix-Socrates tool, http://www.socialcontactdata.org/socrates-comix/.

## Abbreviations

COVID: Coronavirus disease
CoMix: Contact mixing
NBI GAMLSS: Negative binomial generalized additive model for location, scale, and shape
MCAR: Missing completely at random
MAR: Missing at random
MNAR: Missing not at random
NPI: Non-pharmaceutical intervention
UK: United Kingdom
SARS-COV: Severe acute respiratory syndrome coronavirus
GLMM: Generalized linear mixed models
GAMM: Generalized additive mixed models
PI: Pharmaceutical intervention

## References

[1] World Health Organization. BWorld Robot Control Software. 2020. https://www.who.int/news-room/fact-sheets/detail/the-top-10-causes-of-death. Accessed 19 Nov 2021.

[2] Eubank S, Guclu H, Anil Kumar V, Marathe MV, Srinivasan A, Toroczkai Z (2004). Modelling disease outbreaks in realistic urban social networks. Nature..

[3] Wallinga J, Teunis P, Kretzschmar M (2006). Using data on social contacts to estimate age-specific transmission parameters for respiratory-spread infectious agents. Am J Epidemiol..

[4] Hoang T, Coletti P, Melegaro A, Wallinga J, Grijalva CG, Edmunds JW (2019). A systematic review of social contact surveys to inform transmission models of close-contact infections. Epidemiol (Camb Mass)..

[5] Ogunjimi B, Hens N, Goeyvaerts N, Aerts M, Van Damme P, Beutels P (2009). Using empirical social contact data to model person to person infectious disease transmission: an illustration for varicella. Math Biosci..

[6] Hens N, Ayele GM, Goeyvaerts N, Aerts M, Mossong J, Edmunds JW (2009). Estimating the impact of school closure on social mixing behaviour and the transmission of close contact infections in eight European countries. BMC Infect Dis..

[7] Eames K, Tilston NL, White PJ, Adams E, Edmunds W (2010). The impact of illness and the impact of school closure on social contact patterns. Health Technol Assess (Winchester England)..

[8] Liu CY, Berlin J, Kiti MC, Del Fava E, Grow A, Zagheni E (2021). Rapid review of social contact patterns during the COVID-19 pandemic. Epidemiol (Camb Mass)..

[9] Gimma A, Munday JD, Wong KL, Coletti P, van Zandvoort K, Prem K (2022). Changes in social contacts in England during the COVID-19 pandemic between March 2020 and March 2021 as measured by the CoMix survey: A repeated cross-sectional study. PLoS Med..

[10] Coletti P, Wambua J, Gimma A, Willem L, Vercruysse S, Vanhoutte B (2020). CoMix: comparing mixing patterns in the Belgian population during and after lockdown. Sci Rep..

[11] Zhang J, Litvinova M, Liang Y, Wang Y, Wang W, Zhao S (2020). Changes in contact patterns shape the dynamics of the COVID-19 outbreak in China. Science..

[12] Jarvis CI, Van Zandvoort K, Gimma A, Prem K, Klepac P, Rubin GJ (2020). Quantifying the impact of physical distance measures on the transmission of COVID-19 in the UK. BMC Med..

[13] Backer JA, Mollema L, Vos ER, Klinkenberg D, Van Der Klis FR, De Melker HE (2021). Impact of physical distancing measures against COVID-19 on contacts and mixing patterns: repeated cross-sectional surveys, the Netherlands, 2016–17, April 2020 and June 2020. Eurosurveillance..

[14] Latsuzbaia A, Herold M, Bertemes JP, Mossong J (2020). Evolving social contact patterns during the COVID-19 crisis in Luxembourg. PloS ONE..

[15] Gimma A, Munday JD, Wong KL, Coletti P, van Zandvoort K, Prem K, Jarvis CI. Changes in social contacts in England during the COVID-19 pandemic between March 2020 and March 2021 as measured by the CoMix survey: A repeated cross-sectional study. PLoS Med. 2022;19(3):e1003907. 10.1371/journal.pmed.1003907. 10.1371/journal.pmed.1003907 PMC8887739 35231023

[16] Verelst F, Hermans L, Vercruysse S, Gimma A, Coletti P, Backer JA (2021). SOCRATES-CoMix: a platform for timely and open-source contact mixing data during and in between COVID-19 surges and interventions in over 20 European countries. BMC Med..

[17] Wong KL, Gimma A, Paixao ES, Faes C, Beutels P, Hens N, Edmunds WJ. Pregnancy during COVID-19: social contact patterns and vaccine coverage of pregnant women from CoMix in 19 European countries. BMC Pregnancy Childbirth. 2022;22(1):1-12. 10.1186/s12884-022-05076-1. 10.1186/s12884-022-05076-1 PMC9547635 36209078

[18] Drolet M, Godbout A, Mondor M, Béraud G, Drolet-Roy L, Lemieux-Mellouki P (2022). Time trends in social contacts before and during the COVID-19 pandemic: the CONNECT study. BMC Publ Health..

[19] Trentini F, Guzzetta G, Galli M, Zardini A, Manenti F, Putoto G (2021). Modeling the interplay between demography, social contact patterns, and SARS-CoV-2 transmission in the South West Shewa Zone of Oromia Region, Ethiopia. BMC Med..

[20] Quaife M, Van Zandvoort K, Gimma A, Shah K, McCreesh N, Prem K (2020). The impact of COVID-19 control measures on social contacts and transmission in Kenyan informal settlements. BMC Med..

[21] Zhang J, Litvinova M, Liang Y, Zheng W, Shi H, Vespignani A, et al. The impact of relaxing interventions on human contact patterns and SARS-CoV-2 transmission in China. Sci Adv. 2021;7(19):eabe2584. 10.1126/sciadv.abe2584 PMC8104862 33962957

[22] Egleston BL, Miller SM, Meropol NJ (2011). The impact of misclassification due to survey response fatigue on estimation and identifiability of treatment effects. Stat Med..

[23] Lavrakas, P. J. (Ed.). Encyclopedia of survey research methods. SAGE Publications, Inc. 2008. 10.4135/9781412963947.

[24] Engel U (2015). Survey measurements: techniques, data quality and sources of error.

[25] Neter J, Waksberg J (1964). A study of response errors in expenditures data from household interviews. J Am Stat Assoc..

[26] Backor K, Golde S, Nie N. Estimating survey fatigue in time use study. In: International Association for Time Use Research Conference. Washington, DC: Citeseer; 2007.

[27] Molenberghs G, Verbeke G. Models for discrete longitudinal data. New York: Springer; 2005.

[28] Sinickas A (2007). Finding a cure for survey fatigue. Strateg Commun Manag..

[29] Pecoraro J (2012). Survey fatigue. Qual Prog..

[30] Held L, Hens N, D O’Neill P, Wallinga J (2019). Handbook of infectious disease data analysis.

[31] Rigby RA, Stasinopoulos DM (2005). Generalized additive models for location, scale and shape. J R Stat Soc Ser C (Appl Stat)..

[32] Stasinopoulos DM, Rigby RA (2008). Generalized additive models for location scale and shape (GAMLSS) in R. J Stat Softw..

[33] Diggle P, Diggle PJ, Heagerty P, Liang KY, Zeger S (2002). Analysis of longitudinal data.

[34] Rigby B, Stasinopoulos M. A Flexible Regression Approach Using GAMLSS in R. 2010. Techinal Report.

[35] Molenberghs G, Verbeke G, Demétrio CG (2007). An extended random-effects approach to modeling repeated, overdispersed count data. Lifetime Data Anal..

[36] Molenberghs G, Verbeke G, Demétrio CG, Vieira AM (2010). A family of generalized linear models for repeated measures with normal and conjugate random effects. Stat Sci..

[37] Mossong J, Hens N, Jit M, Beutels P, Auranen K, Mikolajczyk R (2008). Social contacts and mixing patterns relevant to the spread of infectious diseases. PLoS Med..

[38] Van Hoang T, Coletti P, Kifle YW, Van Kerckhove K, Vercruysse S, Willem L (2021). Close contact infection dynamics over time: insights from a second large-scale social contact survey in Flanders, Belgium, in 2010–2011. BMC Infect Dis..

[39] Hastie T, Tibshirani R (1993). Varying-coefficient models. J R Stat Soc Ser B (Methodol)..

[40] Reinsch CH (1967). Smoothing by spline functions. Numer Math..

[41] Green PJ, Silverman BW (1993). Nonparametric regression and generalized linear models: a roughness penalty approach.

[42] Hastie T, Tibshirani R. Generalized additive models london chapman and hall. Inc. 1990. 10.1201/9780203753781.

[43] FFunk S, Willem L. socialmixr: Social Mixing Matrices for Infectious Disease Modelling. R package version 0.2.0. 2022.

[44] Population Division, Department of Economic and Social Affairs, United Nations. wpp2015: World population prospects 2015. The Comprehensive R Archive Network. 2019.

[45] Willem L, Van Hoang T, Funk S, Coletti P, Beutels P, Hens N (2020). SOCRATES: an online tool leveraging a social contact data sharing initiative to assess mitigation strategies for COVID-19. BMC Res Notes..

[46] Willem L, Van Kerckhove K, Chao DL, Hens N, Beutels P (2012). A nice day for an infection? Weather conditions and social contact patterns relevant to influenza transmission. PLoS ONE..

[47] Rubin D (1976). Inference and missing data. Biometrika..

[48] Molenberghs G, Thijs H, Jansen I, Beunckens C, Kenward MG, Mallinckrodt C (2004). Analyzing incomplete longitudinal clinical trial data. Biostatistics..

[49] Wood SN. Generalized Additive Models: an Introduction with R. Florida: CRC Press; 2017.

[50] Diekmann O, Heesterbeek JAP, Metz JA (1990). On the definition and the computation of the basic reproduction ratio R 0 in models for infectious diseases in heterogeneous populations. J Math Biol..

[51] Franco N, Coletti P, Willem L, Angeli L, Lajot A, Abrams S (2022). Inferring age-specific differences in susceptibility to and infectiousness upon SARS-CoV-2 infection based on Belgian social contact data. PLoS Comput Biol..

[52] Sciensano. the Belgian public health institute. https://epistat.wiv-isp.be/covid/. Accessed 29 June 2022.

[53] Gressani O, Wallinga J, Althaus CL, Hens N, Faes C. EpiLPS: A fast and flexible Bayesian tool for estimation of the time-varying reproduction number. PLoS Comput Biol. 2022;18(10):e1010618. 10.1371/journal.pcbi.1010618. 10.1371/journal.pcbi.1010618 PMC9584461 36215319

[54] Faes C, Abrams S, Van Beckhoven D, Meyfroidt G, Vlieghe E, Hens N. Belgian Collaborative Group on COVID-19 Hospital Surveillance. Time between symptom onset, hospitalisation and recovery or death: Statistical analysis of belgian covid-19 patients. Int J Environ Res Public Health. 2020;17(20):7560. 10.3390/ijerph17207560 PMC7589278 33080869

[55] Diekmann O, Heesterbeek H, Britton T (2013). Mathematical tools for understanding infectious disease dynamics.

[56] Wallinga J, van Boven M, Lipsitch M (2010). Optimizing infectious disease interventions during an emerging epidemic. Proc Natl Acad Sci..

[57] Prem K, Liu Y, Russell TW, Kucharski AJ, Eggo RM, Davies N (2020). The effect of control strategies to reduce social mixing on outcomes of the COVID-19 epidemic in Wuhan, China: a modelling study. Lancet Publ Health..

[58] Hale T, Angrist N, Goldszmidt R, Kira B, Petherick A, Phillips T (2021). A global panel database of pandemic policies (Oxford COVID-19 Government Response Tracker). Nat Hum Behav..

[59] Federal Public Service (FPS) Health FCS, Environment. Corona virus COVID-19 measurements. 2021. https://www.info-coronavirus.be/en/news/. Accessed 30 Apr 2023.

[60] Marathe A, Lewis B, Chen J, Eubank S (2011). Sensitivity of household transmission to household contact structure and size. PLoS ONE..

[61] Wambua J, Hermans L, Coletti P, Verelst F, Willem L, Jarvis CI (2022). The influence of risk perceptions on close contact frequency during the SARS-CoV-2 pandemic. Sci Rep..

[62] Goeyvaerts N, Santermans E, Potter G, Torneri A, Van Kerckhove K, Willem L (1893). Household members do not contact each other at random: implications for infectious disease modelling. Proc R Soc B..

[63] O’Reilly-Shah VN (2017). Factors influencing healthcare provider respondent fatigue answering a globally administered in-app survey. PeerJ..

[64] Little R (1995). Modeling dropout mechanism for multivariate incomplete data. J Am Stat Assoc..

[65] Kenward MG, Carpenter J (2007). Multiple imputation: current perspectives. Stat Methods Med Res..

[66] Backer JA, Bogaardt L, Beutels P, Coletti P, Edmunds WJ, Gimma A (2023). Dynamics of non-household contacts during the COVID-19 pandemic in 2020 and 2021 in the Netherlands. Sci Rep..

[67] Google. COVID-19 Community Mobility Reports. 2023. www.google.com/covid19/mobility/. Accessed 18 Apr 2023.

[68] Davies NG, Abbott S, Barnard RC, Jarvis CI, Kucharski AJ, Munday JD (2021). Estimated transmissibility and impact of SARS-CoV-2 lineage B. 1.1. 7 in England. Science..

[69] Grantz KH, Meredith HR, Cummings DA, Metcalf CJE, Grenfell BT, Giles JR (2020). The use of mobile phone data to inform analysis of COVID-19 pandemic epidemiology. Nat Commun..

[70] Kennedy EB, Charifson M, Jehn M, Jensen EA, Vikse J (2022). Prospective sampling bias in COVID-19 recruitment methods: experimental evidence from a national randomized survey testing recruitment materials. BMC Med Res Methodol..

[71] Joyal-Desmarais K, Stojanovic J, Kennedy EB, Enticott JC, Boucher VG, Vo H, et al. How well do covariates perform when adjusting for sampling bias in online COVID-19 research? Insights from multiverse analyses. Eur J Epidemiol. 2022;1–18. 10.1007/s10654-022-00932-y PMC9638233 36335560

[72] De Man J, Campbell L, Tabana H, Wouters E (2021). The pandemic of online research in times of COVID-19. BMJ Open..

[73] Fernández-Sanlés A, Smith D, Clayton GL, Northstone K, Carter AR, Millard LA (2022). Bias from questionnaire invitation and response in COVID-19 research: an example using ALSPAC. Wellcome Open Res..

[74] Coronavirus: reinforced measures. https://www.belgium.be/en/news/2020/coronavirus_reinforced_measures. Accessed 14 June 2023.

[75] Measures taken by the National Security Council of 6 May 2020. https://www.belgium.be/en/news/2020/measures_taken_national_security_council_6_may_2020. Accessed 14 June 2023.

[76] Cheat Sheet: The ‘Easter pause’ rules. https://www.brusselstimes.com/161624/cheat-sheet-the-easter-pause-rules-consultative-committee-alexander-de-croo-hairdressers-shops-coronavirus-crisis-lockdown-hospitals. Accessed 14 June 2023.

[77] Wolter F, Mayerl J, Andersen HK, Wieland T, Junkermann J (2022). Overestimation of COVID-19 Vaccination Coverage in Population Surveys Due to Social Desirability Bias: Results of an Experimental Methods Study in Germany. Socius..

[78] Krumpal I (2013). Determinants of social desirability bias in sensitive surveys: a literature review. Qual Quant..

[79] Tourangeau R, Yan T (2007). Sensitive questions in surveys. Psychol Bull..

[80] Bruyndonckx R, Aerts M, Hens N (2016). Simulation-based evaluation of the performance of the F test in a linear multilevel model setting with sparseness at the level of the primary unit. Biom J..

[81] Bruyndonckx R, Hens N, Aerts M (2018). Simulation-based evaluation of the linear-mixed model in the presence of an increasing proportion of singletons. Biom J..

[82] Maas CJ, Hox JJ (2005). Sufficient sample sizes for multilevel modeling. Methodol Eur J Res Methods Behav Soc Sci..

[83] Clarke P (2008). When can group level clustering be ignored? Multilevel models versus single-level models with sparse data. J Epidemiol Community Health..

[84] Lee OE, Braun TM (2012). Permutation tests for random effects in linear mixed models. Biometrics..

[85] Coletti P, Wambua J, Gimma A, Willem L, Vercruysse S, Vanhoutte B, et al. CoMix social contact data (Belgium). Zenodo. 2020. 10.5281/zenodo.7086043.

