# Supplementary material for: Longitudinal social contact data analysis: insights from 2 years of data collection in Belgium during the COVID-19 pandemic
Source: BMC Public Health. 2023 Jul 6;23:1298. doi: 10.1186/s12889-023-16193-7 (PMC10326964; doi:10.1186/s12889-023-16193-7)
Supplement: Supplementary file 1 — Additional file 1. Supporting information. This additional file provides supporting information to support the findings on the main article. [file 12889_2023_16193_MOESM1_ESM.pdf]

Additional file 1 for: "Longitudinal social contact data analysis: insights from 2 years of data collection in Belgium during the COVID-19 pandemic"

Neilshan Loedy[1*], Pietro Coletti[1], James Wambua[1], Lisa Hermans[1], Lander Willem[2],Christopher I. Jarvis[3], Kerry L.M. Wong[3], W. John Edmunds[3], Alexis Robert [3], Quentin J. Leclerc[3,4,7], Ammy Gimma[3], Geert Molenberghs[1,6], Philippe Beutels[2,5], Christel Faes[1], Niel Hens[1,2]

[1]Data Science Institute, I-BioStat, Hasselt University, Hasselt, Belgium.

[2]Centre for Health Economics Research and Modelling Infectious Diseases, Vaccine & Infectious Disease Institute, University of Antwerp, Antwerp, Belgium.

[3]Centre for Mathematical Modelling of Infectious Diseases, Department of Infectious Disease Epidemiology, Faculty of Epidemiology Population Health, London School of Hygiene Tropical Medicine, London, United Kingdom.

[4]Department of Infectious Disease Epidemiology, Faculty of Epidemiology and Public Health, London School of Hygiene Tropical Medicine, London, United Kingdom. [5]School of Public Health and Community Medicine, The University of New South Wales, Sydney, Australia.

[6]L-BioStat, Department of Public Health and Primary Care, Faculty of Medicine, KU Leuven, Leuven, Belgium.

[7]Epidemiology and modelling of bacterial escape to antimicrobials, Institut Pasteur, Paris, France.

* Corresponding author: neilshan.loedy@uhasselt.be

1. Additional file 1 : Supporting information

This additional file provides supporting information to support the findings on the main article.

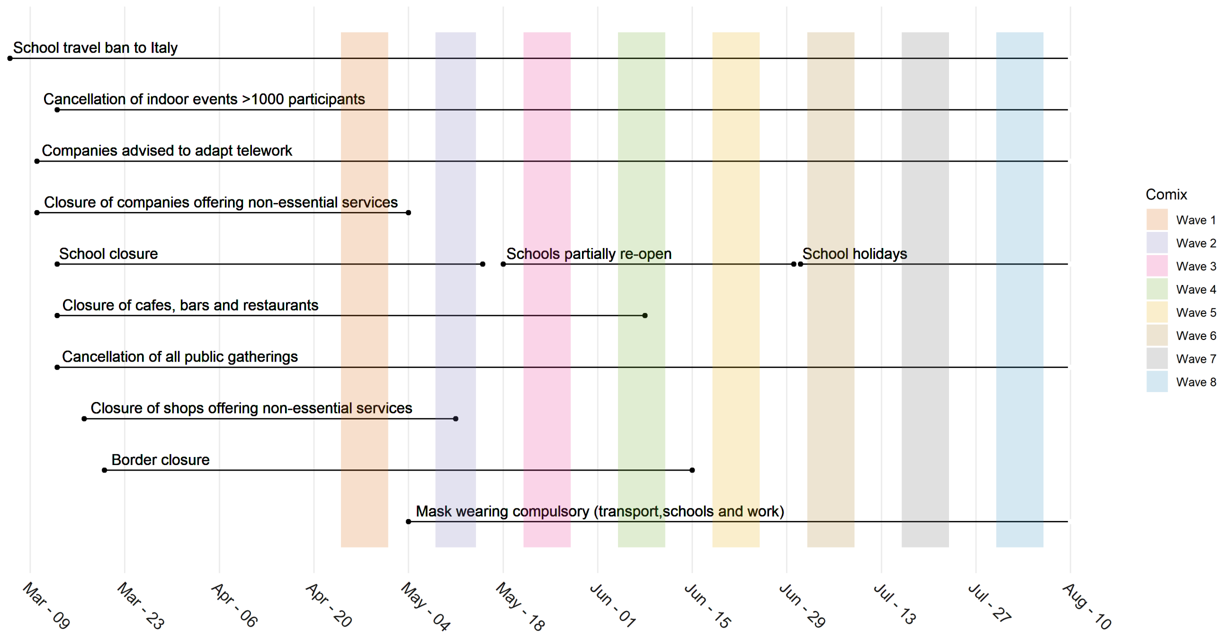

Figure S1: Calendar of non-pharmaceutical interventions (NPIs) and CoMix waves for the first survey wave (waves 1–8) [1]

Table S1: Description of the parameters

Parameter	Description	Variable
Household size	The number of people who live at the same address and with whom the participant shares a kitchen.	Elderly (1, 2, 3+), Adults (1, 2, 3, 4+), Children (2, 3, 4+)
Area	Region of residency	Brussels, Flemish, Wallonia
Wave bin	Number of participation on the survey	1, 2, 3, . . . , 7, 8+
Elevated risk	The condition when participants have a chronic respiratory disease, chronic heart condition, chronic kidney disease, chronic liver disease, chronic neurological disease, diabetes (any types), a weakened immune system (due to disease or medication), asplenia or malfunctioning spleen, morbid obesity (BMI $\geq$ 40), and pregnant women.	Yes, No
Weekend	Saturday or Sunday	Weekday, Weekend
Holiday	Belgium nationally recognized non-working day when most business and institutions are closed	Yes, No
Face mask	The usage of face masks when participants are doing the reported contacts.	Yes, No
Vaccination status	Vaccination conditions of participants for at least one injection of the vaccine	Yes, No
Symptomatic status	The condition when participants have a fever or high temperature, a cough that has lasted for at least several hours, shortness of breath, aches in arms or legs, blocked nose, sore throat, or feeling tired.	Yes, No

Table S2: Summary of survey waves

Wave	Period	Wave	Period	Wave	Period
09	12 Nov - 19 Nov'20	21	27 Apr - 03 May'21	33	12 Oct - 17 Oct'21
10	27 Nov - 09 Dec'20	22	12 May - 19 May'21	34	27 Oct - 03 Nov'21
11	10 Dec - 17 Dec'20	23	25 May - 01 Jun'21	35	09 Nov - 15 Nov'21
12	22 Dec - 04 Jan'21	24	09 Jun - 16 Jun'21	36	23 Nov - 29 Nov'21
13	05 Jan - 11 Jan'21	25	22 Jun - 27 Jun'21	37	07 Dec - 13 Dec'21
14	19 Jan - 24 Jan'21	26	06 Jul - 14 Jul'21	38	21 Dec - 28 Dec'21
15	02 Feb - 07 Feb'21	27	20 Jul - 26 Jul'21	39	04 Jan - 11 Jan'22
16	16 Feb - 23 Feb'21	28	03 Aug - 10 Aug'21	40	18 Jan - 23 Jan'22
17	02 Mar - 09 Mar'21	29	17 Aug - 23 Aug'21	41	01 Feb - 08 Feb'22
18	16 Mar - 23 Mar'21	30	31 Aug - 07 Sep'21	42	16 Feb - 22 Feb'22
19	30 Mar - 06 Apr'21	31	14 Sep - 20 Sep'21	43	01 Mar - 08 Mar'22
20	13 Apr - 19 Apr'21	32	28 Sep - 04 Oct'21		

Table S3: Summary of CoMix survey. The percentages are rounded to two decimal place and thus might not add up to 100%

Waves of participation	1	2	3	4	5	6	7	8+
All	7356	4616	3606	3156	2897	2632	2469	28192
**Participants age**								
Elderly	366 (4.98%)	275 (5.96%)	249 (6.91%)	258 (8.17%)	254 (8.77%)	244 (9.27%)	245 (9.92%)	3528 (12.51%)
Adults	4462 (60.66%)	2779 (60.20%)	2214 (61.40%)	2050 (64.96%)	1912 (66.00%)	1714 (65.12%)	1605 (65.01%)	18901 (67.04%)
Children	2528 (34.37%)	1562 (33.84%)	1143 (31.70%)	848 (26.87%)	731 (25.23%)	674 (25.61%)	619 (25.07%)	5763 (20.44%)
**Contacts age**								
Elderly	1382 (18.79%)	928 (20.10%)	796 (22.07%)	878 (27.82%)	832 (28.72%)	764 (29.03%)	743 (30.09%)	9212 (32.68%)
Adults	4206 (57.18%)	2713 (58.77%)	2141 (59.37%)	1725 (54.66%)	1573 (54.30%)	1429 (54.29%)	1336 (54.11%)	15020 (53.28%)
Children	1768 (24.03%)	975 (21.12%)	669 (18.55%)	553 (17.52%)	492 (16.98%)	439 (16.68%)	390 (15.80%)	3960 (14.05%)
**Weekday/Weekend**								
Weekday	6946 (94.43%)	3840 (83.19%)	2956 (81.97%)	2576 (81.62%)	2191 (75.63%)	1921 (72.99%)	1818 (73.63%)	19520 (69.24%)
Weekend	410 (5.57%)	776 (16.81%)	650 (18.03%)	580 (18.38%)	706 (24.37%)	711 (27.01%)	651 (26.37%)	8672 (30.76%)
**Area**								
Brussels Central Region	640 (8.70%)	433 (9.38%)	298 (8.26%)	259 (8.21%)	237 (8.18%)	194 (7.37%)	199 (8.06%)	1936 (6.87%)
Flemish Region	4141 (56.29%)	2617 (56.69%)	2064 (57.24%)	1864 (59.06%)	1746 (60.27%)	1604 (60.94%)	1479 (59.90%)	17985 (63.79%)
Wallonia Region	2575 (35.01%)	1566 (33.93%)	1244 (34.50%)	1033 (32.73%)	914 (31.55%)	834 (31.69%)	791 (32.04%)	8271 (29.34%)
**Holiday**								
Holiday (Y)	1947 (26.47%)	956 (20.71%)	956 (20.71%)	1892 (59.95%)	804 (27.75%)	757 (28.76%)	745 (30.17%)	8653 (30.69%)
Holiday (N)	5409 (73.53%)	3660 (79.29%)	2610 (72.38%)	1264 (40.05%)	2093 (72.25%)	1875 (71.24%)	1724 (69.83%)	19539 (69.31%)
**Household size**								
1	832 (11.31%)	596 (12.91%)	530 (14.70%)	477 (15.11%)	445 (15.36%)	387 (14.70%)	357 (14.46%)	3343 (11.86%)
2	1943 (26.41%)	1393 (30.18%)	1173 (32.53%)	1122 (35.55%)	1062 (36.66%)	987 (37.50%)	956 (38.72%)	12654 (44.89%)
3	1694 (23.03%)	974 (21.10%)	741 (20.55%)	639 (20.25%)	571 (19.71%)	525 (19.95%)	488 (19.77%)	5716 (20.28%)
4+	2887 (39.25%)	1653 (35.81%)	1162 (32.22%)	918 (29.09%)	819 (28.27%)	733 (27.85%)	668 (27.06%)	6479 (22.98%)
**Elevated risk**								
Yes	1328 (18.05%)	911 (19.74%)	730 (20.24%)	675 (21.39%)	641 (22.13%)	609 (23.14%)	575 (23.29%)	7949 (28.20%)
No	6028 (81.95%)	3705 (80.26%)	2876 (79.76%)	2481 (78.61%)	2256 (77.87%)	2023 (76.86%)	1894 (76.71%)	20243 (71.80%)
**Face mask usage**								
Yes	2712 (63.13%)	2908 (63.00%)	2295 (63.64%)	2064 (65.43%)	1872 (64.62%)	1640 (62.31%)	1506 (61.00%)	17350 (61.54%)
No	4644 (36.87%)	1708 (37.00%)	1311 (34.57%)	1091 (34.57%)	1025 (35.38%)	992 (37.67%)	963 (39.00%)	10842 (38.46%)
**Symptomatic status**								
Yes	4222 (57.40%)	2559 (55.44%)	1847 (51.22%)	1490 (47.21%)	1334 (46.05%)	1212 (46.05%)	1096 (44.39%)	10793 (38.28%)
No	3134 (42.60%)	2057 (44.56%)	1759 (48.78%)	1666 (52.79%)	1563 (53.95%)	1420 (53.95%)	1373 (55.61%)	17399 (61.72%)
**Vaccination status**								
Yes	2292 (31.16%)	964 (20.88%)	675 (18.72%)	640 (20.28%)	627 (21.64%)	637 (24.20%)	698 (28.27%)	15425 (54.71%)
No	3167 (43.05%)	2341 (50.71%)	1891 (52.44%)	1615 (51.17%)	1485 (51.26%)	1287 (48.90%)	1116 (45.20%)	6804 (24.13%)
NA's	1897 (25.79%)	1311 (28.40%)	1040 (28.84%)	901 (28.55%)	785 (27.10%)	785 (26.90%)	655 (26.53%)	5963 (21.15%)

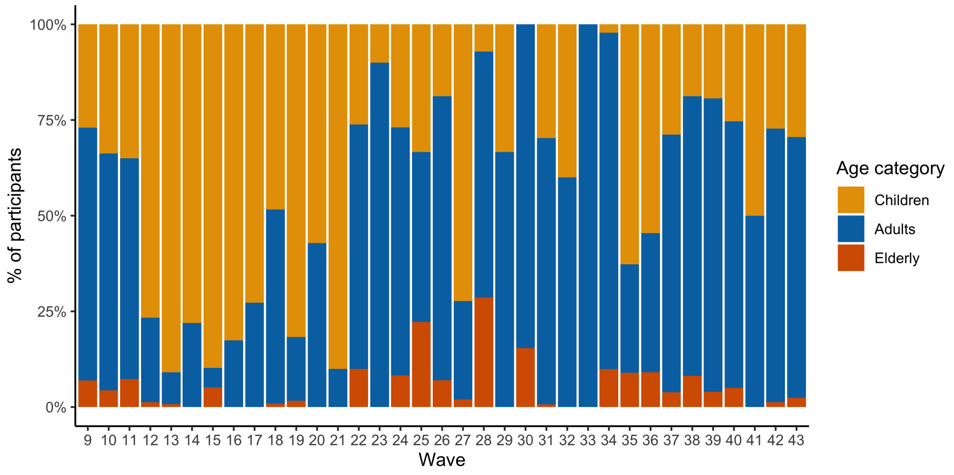

Figure S2: Distribution of age categories of new participants in Belgium CoMix survey

Table S4: Summary of the generalized (pseudo) R^2 for considering SES and Occupation in the NBI GAMLSS.

	Without SES and Occupation	With SES and Occupation	ΔR^2
Elderly	62.19%	62.48%	0.0029
Adults	76.34%	76.58%	0.0024
Children	73.98%	73.48%	0.005

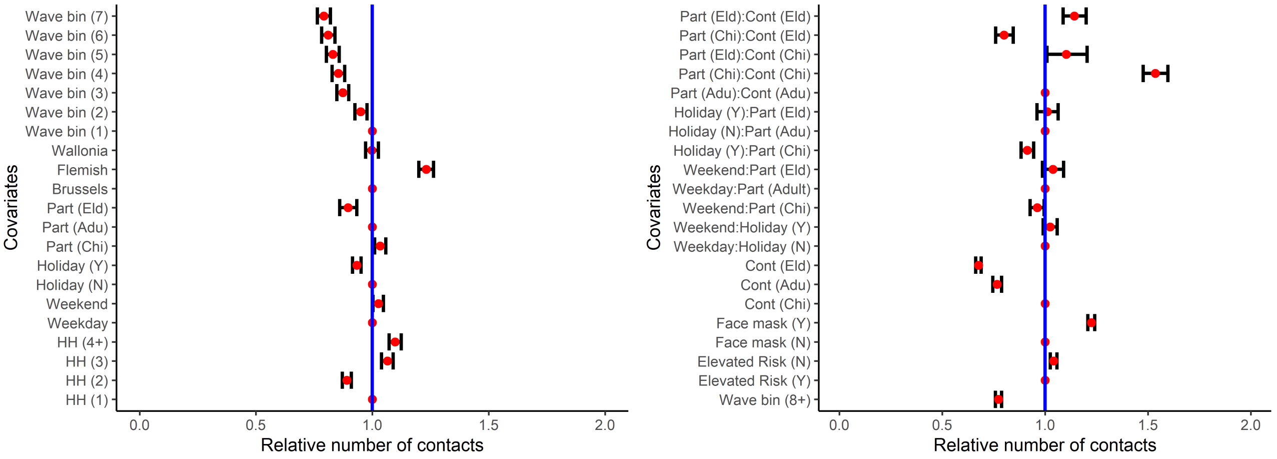

Figure S3: Visualization of mixing pattern between age categories based on relative number of contacts *(red dot)* and 95% confidence interval based on NBI GAMLSS model

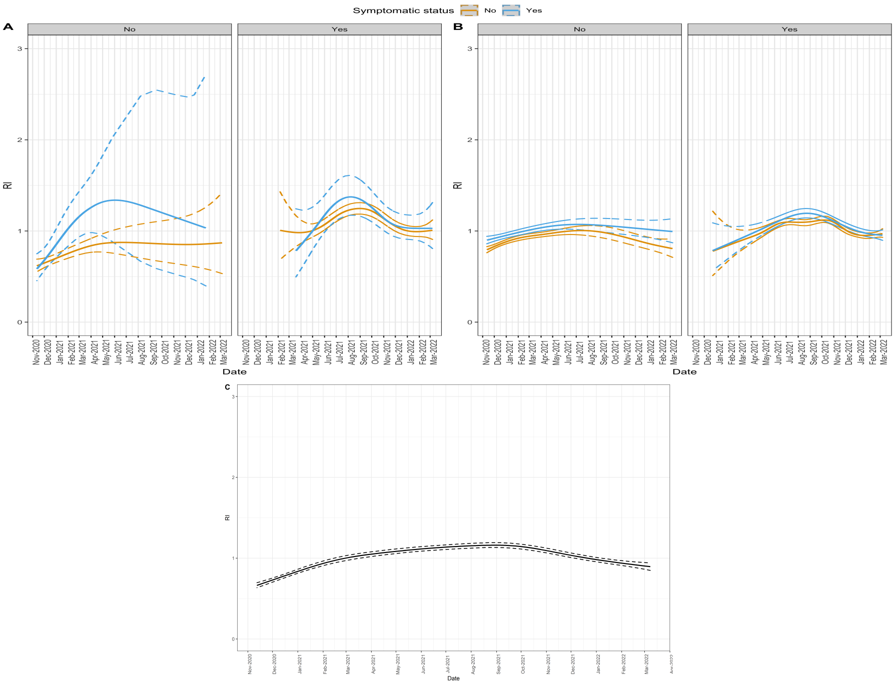

Figure S4: Relative number of contacts and 95% confidence interval based on smoothing parameters on NBI GAMLSS model for different age groups; Penalized Varying Coefficient for *(A) Elderly* and *(B) Adults* (Vaccination status *(box)* and Symptomatic status *(line)*), Cubic Spline for *(C) Children.*

Summary statistics of GAMLSS Model for the variance on Children and Elderly (Table S5 – S6). Note that only random intercept was used to model the variance of GAMLSS Model Adults.

Table S5: Children's GAMLSS model parameter estimates on the variance

Estimate	RI	RI.CI
Area (Flemish Region)	1.5730*	(1.1340,2.1820)
Area (Wallonia Region)	1.1960	(0.8590,1.6660)
Household size (2)	1.2710	(0.8100,1.9950)
Household size (4+)	1.3440	(0.9360,1.9320)
Wave bin (2)	0.9370	(0.8000,1.0950)
Wave bin (3)	0.6750**	(0.5700,0.8010)
Wave bin (4)	0.6990*	(0.5800,0.8420)
Wave bin (5)	0.5320**	(0.4340,0.6520)
Wave bin (6)	0.4590**	(0.3700,0.5700)
Wave bin (7)	0.5430**	(0.4360,0.6760)
Wave bin (8+)	0.3890**	(0.3410,0.4450)
Weekend	0.8030*	(0.7050,0.9130)
Holiday (Y)	0.7330**	(0.6590,0.8150)
Face Mask (Y)	1.0020	(0.9190,1.0920)
Weekend:Holiday (Y)	1.1430	(0.9130,1.4320)
Area (Flemish Region):Household size (2)	0.7400	(0.4520,1.2130)
Area (Wallonia Region):Household size (2)	0.7080	(0.4120,1.2190)
Area (Flemish Region):Household size (4+)	0.5040*	(0.3450,0.7370)
Area (Wallonia Region):Household size (4+)	0.7650	(0.5160,1.1350)

(*)p-value <0.05; (**)p-value <0.0001

Table S6: Elderly's GAMLSS model parameter estimates on the variance

Estimate	RI	RI.CI
Household size (1)	1.8300**	(1.3960,2.3980)
Household size (3+)	0.0030**	(0.0010,0.0050)
Wave bin (2)	0.4580	(0.1790,1.1680)
Wave bin (3)	0.6000	(0.2200,1.6380)
Wave bin (4)	1.0930	(0.4400,2.7110)
Wave bin (5)	0.7680	(0.3140,1.8770)
Wave bin (6)	0.4320	(0.1630,1.1490)
Wave bin (7)	0.3280*	(0.1230,0.8690)
Wave bin (8+)	0.4260	(0.1800,1.0070)
Elevated Risk (N)	0.8670	(0.6730,1.1160)
Weekend	1.0360	(0.7600,1.4120)
Holiday (Y)	0.8950	(0.6300,1.2710)
Face Mask (Y)	0.7070*	(0.5310,0.9410)
Weekend:Holiday (Y)	1.0170	(0.5820,1.7780)

(*)p-value <0.05; (**)p-value <0.0001

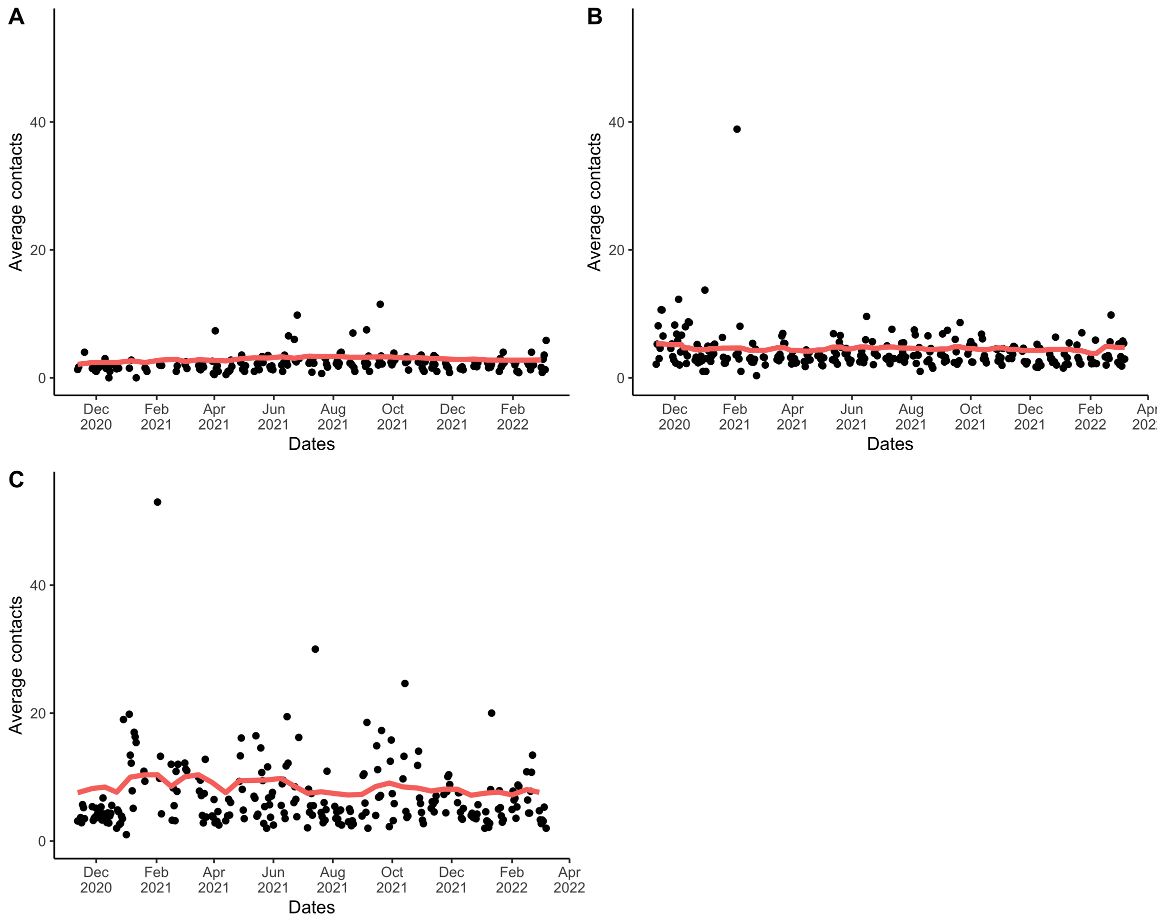

Figure S5: Basic goodness-of-fit plots of GAMLSS NBI for Elderly ($R^2 = 62.19\%$) (A), Adults ($R^2 = 76.34\%$) (B), and Children ($R^2 = 73.98\%$) (C) for the average of number of contacts reported versus the marginal predictions

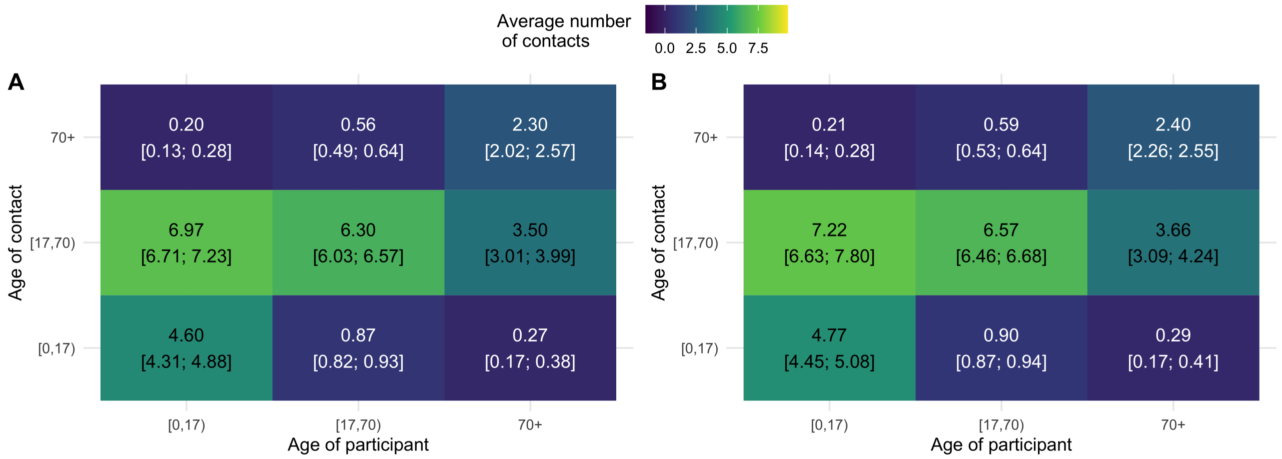

Figure S6: Social contact matrices; average number (wave 9–wave 11) of daily reported contacts *(A) without (B) with under-reporting due to fatigue correction*

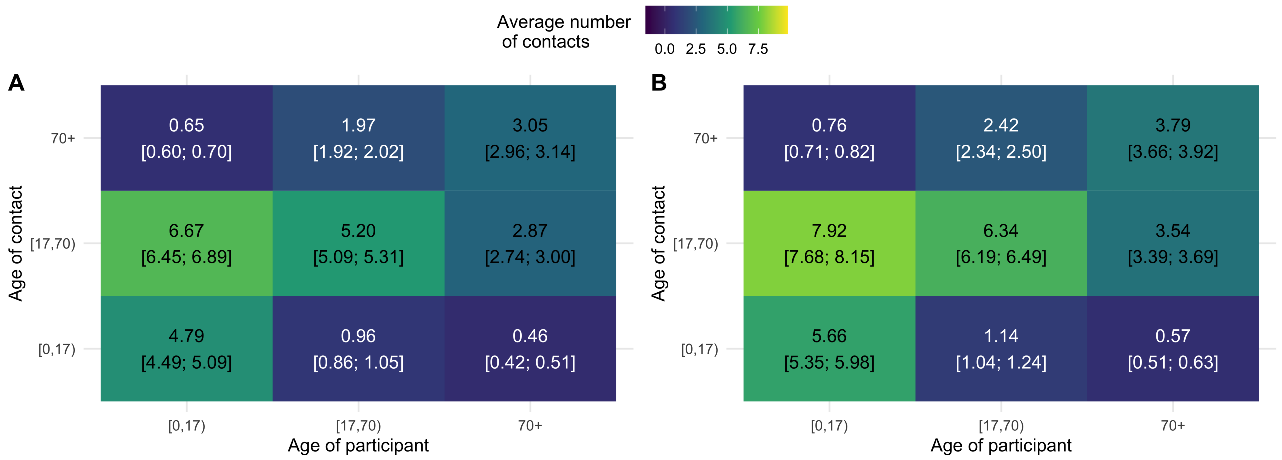

Figure S7: Social contact matrices; average number (wave 12–wave 43) of daily reported contacts *(A) without (B) with under-reporting due to fatigue correction*

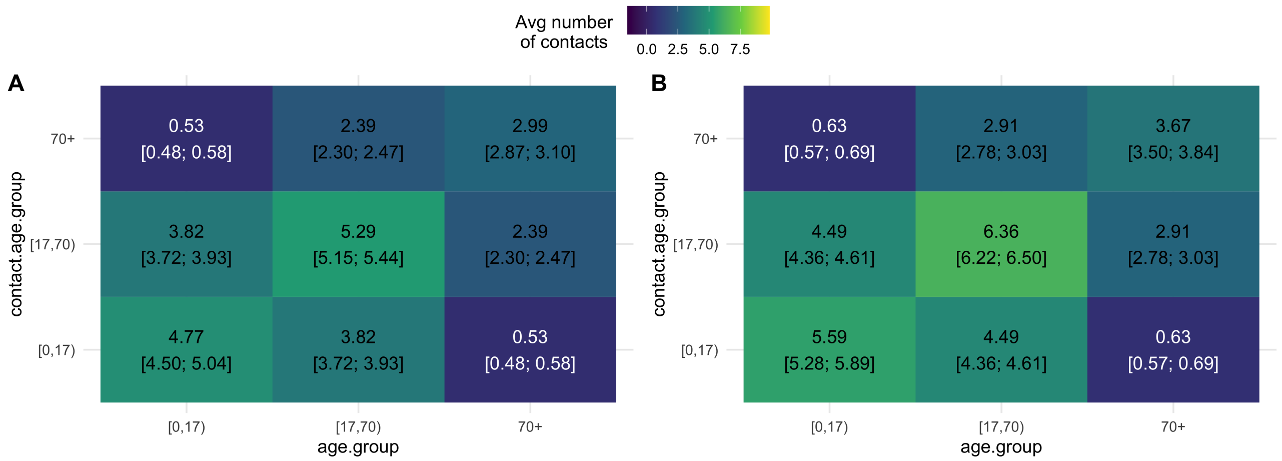

Figure S8: Symmetric social contact matrices taking into account reciprocity in the contact patterns; Average number (wave 9–wave 43) of daily reported contacts *(A) without (B) with under-reporting due to fatigue correction*

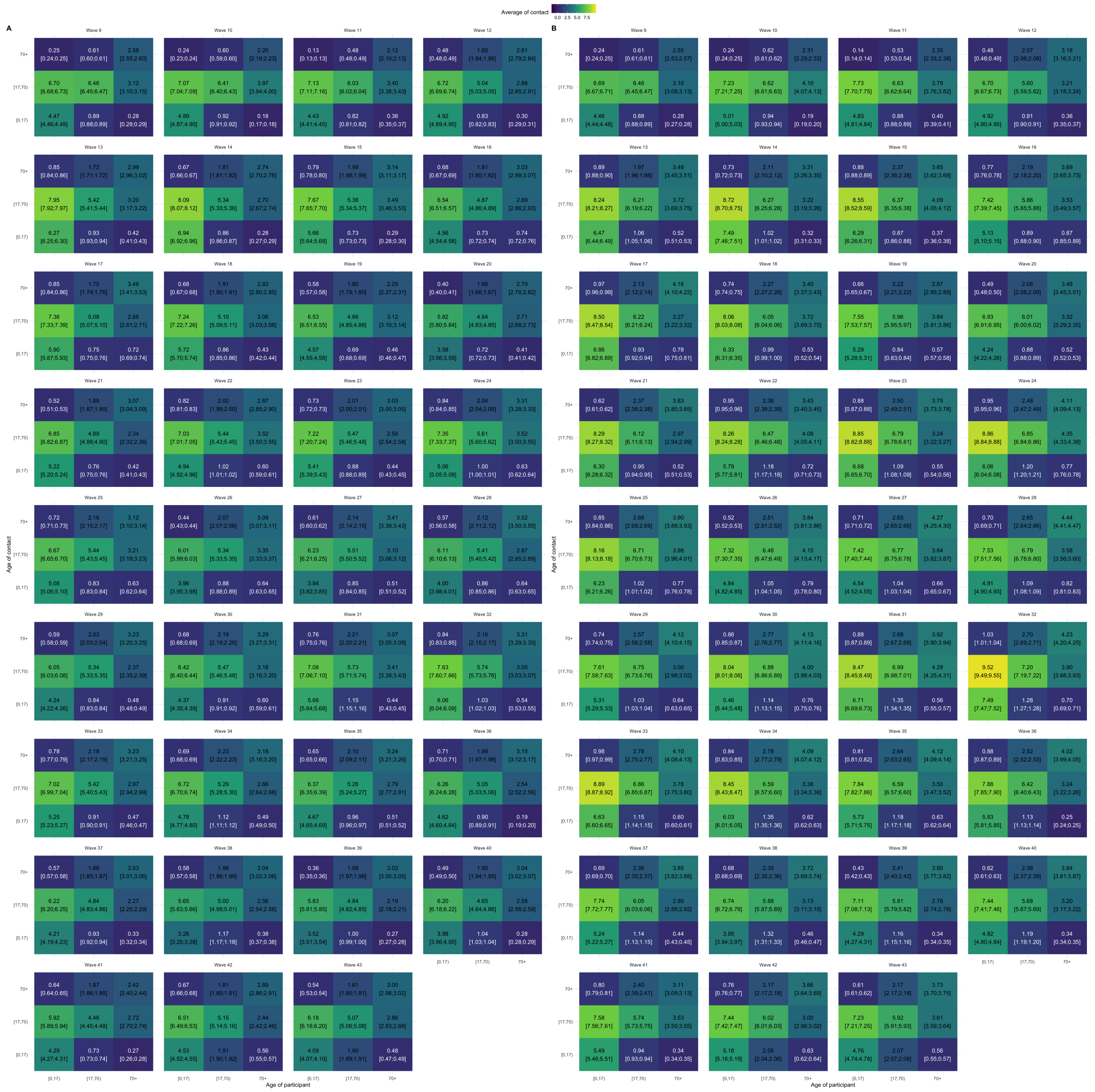

Figure S9: Social contact matrices; average number of daily reported contacts per wave *(A) without fatigue correction. (B) with fatigue correction.*

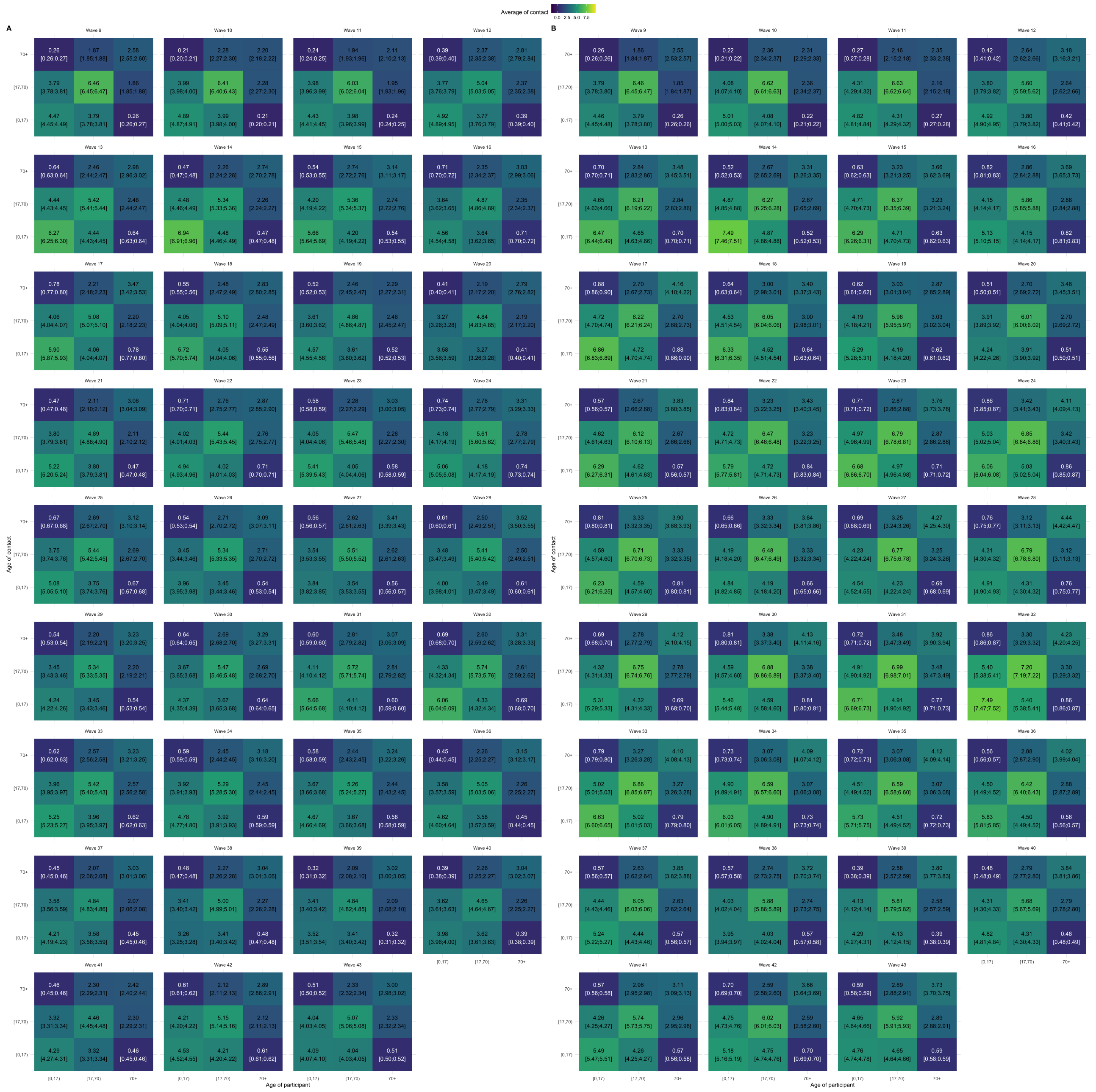

Figure S10: Symmetric social contact matrices; average number of daily reported contacts per wave *(A) without fatigue correction. (B) with fatigue correction.*

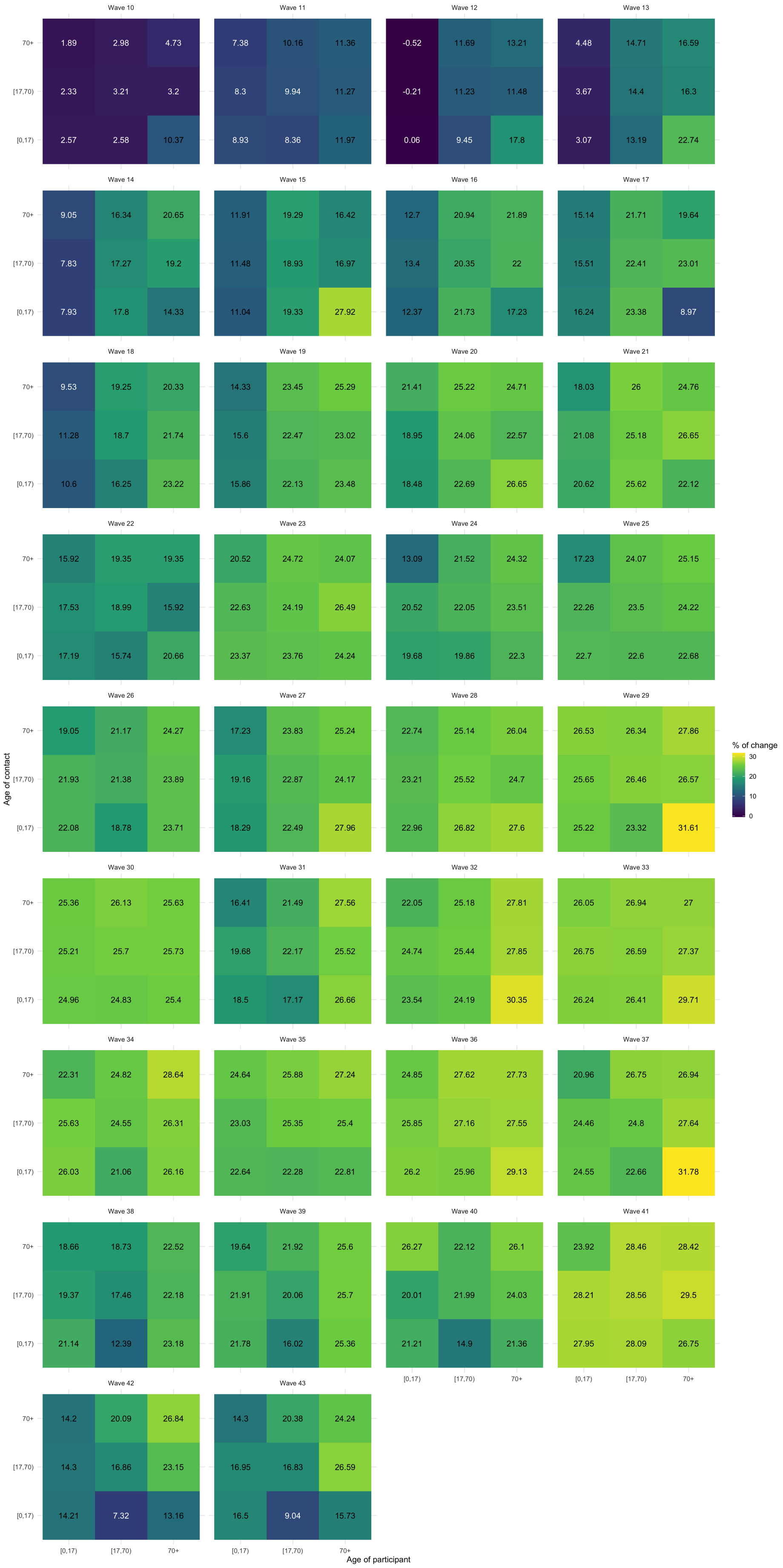

Figure S11: Age-specific percentages of change in average number of contacts by correcting for under-reporting due to fatigue per wave.

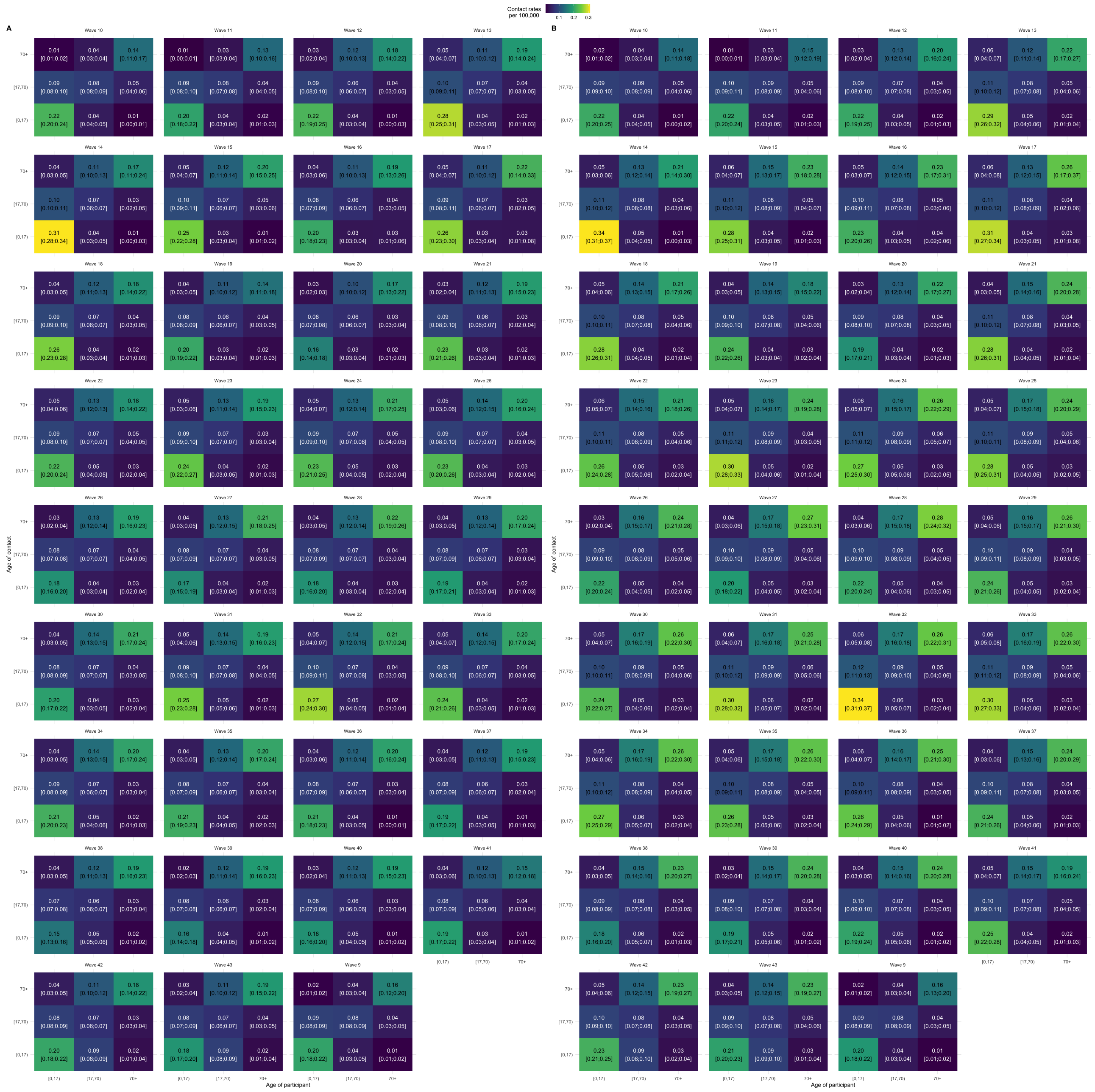

Figure S12: Social contact matrices; average number of contact rates per wave *(A) without fatigue correction. (B) with fatigue correction.*

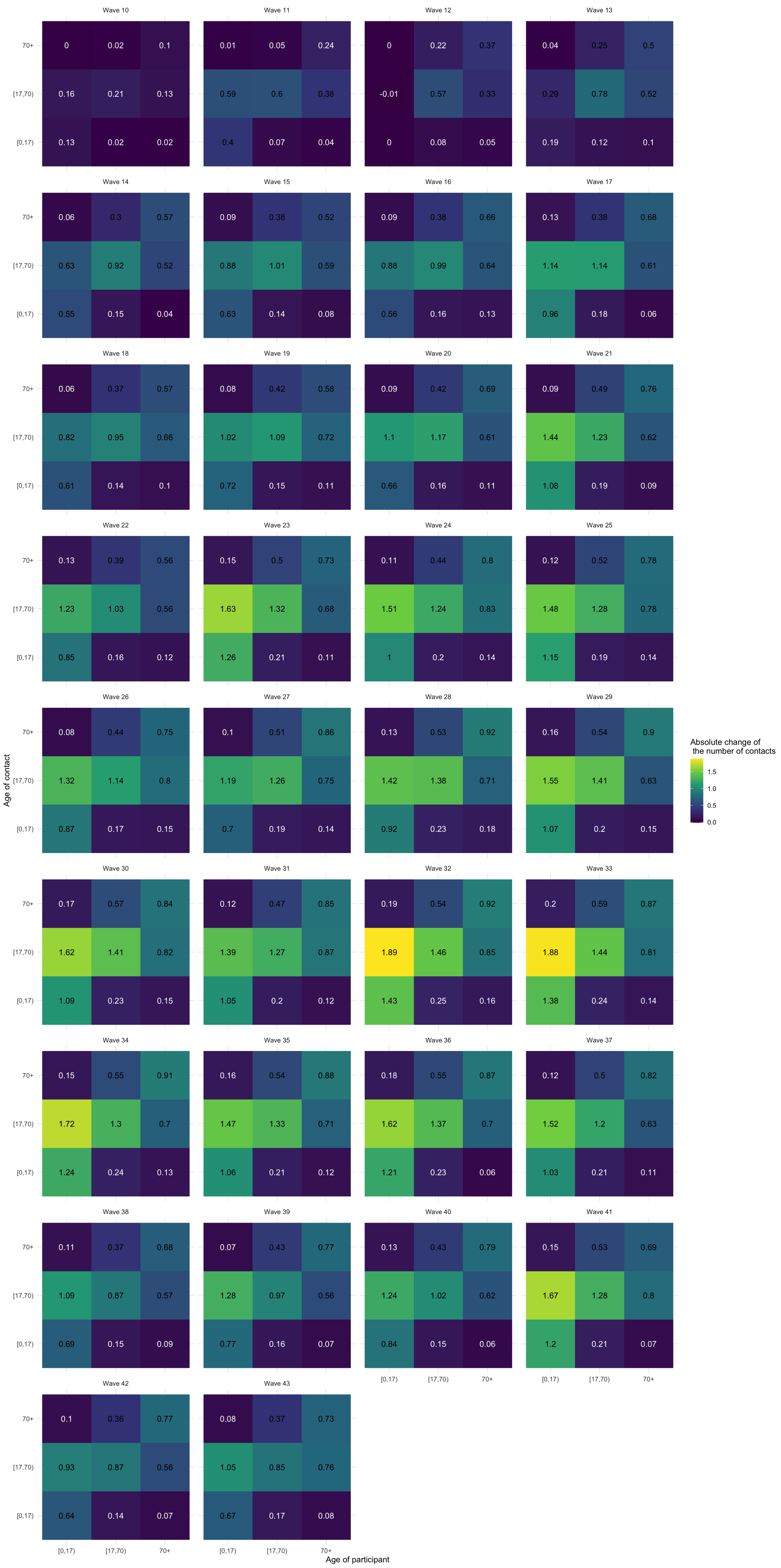

Figure S13: Changes in average number of contacts for each wave with and without fatigue correction.

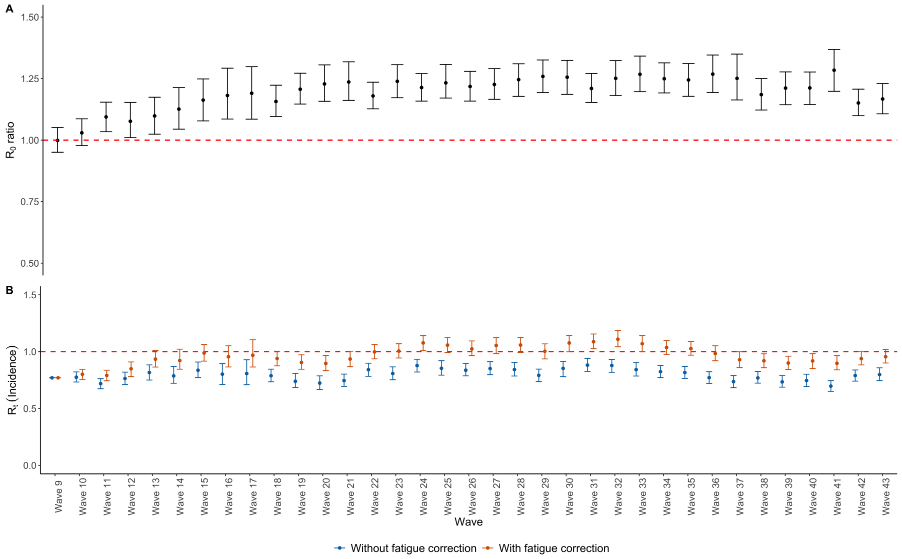

Figure S14: *(A)* Relative changes in R_0 with and without correcting for under-reporting due to fatigue and *(B)* The impact of correcting for under-reporting due to fatigue on the incidence reproduction number estimated from heterogeneous CoMix data.

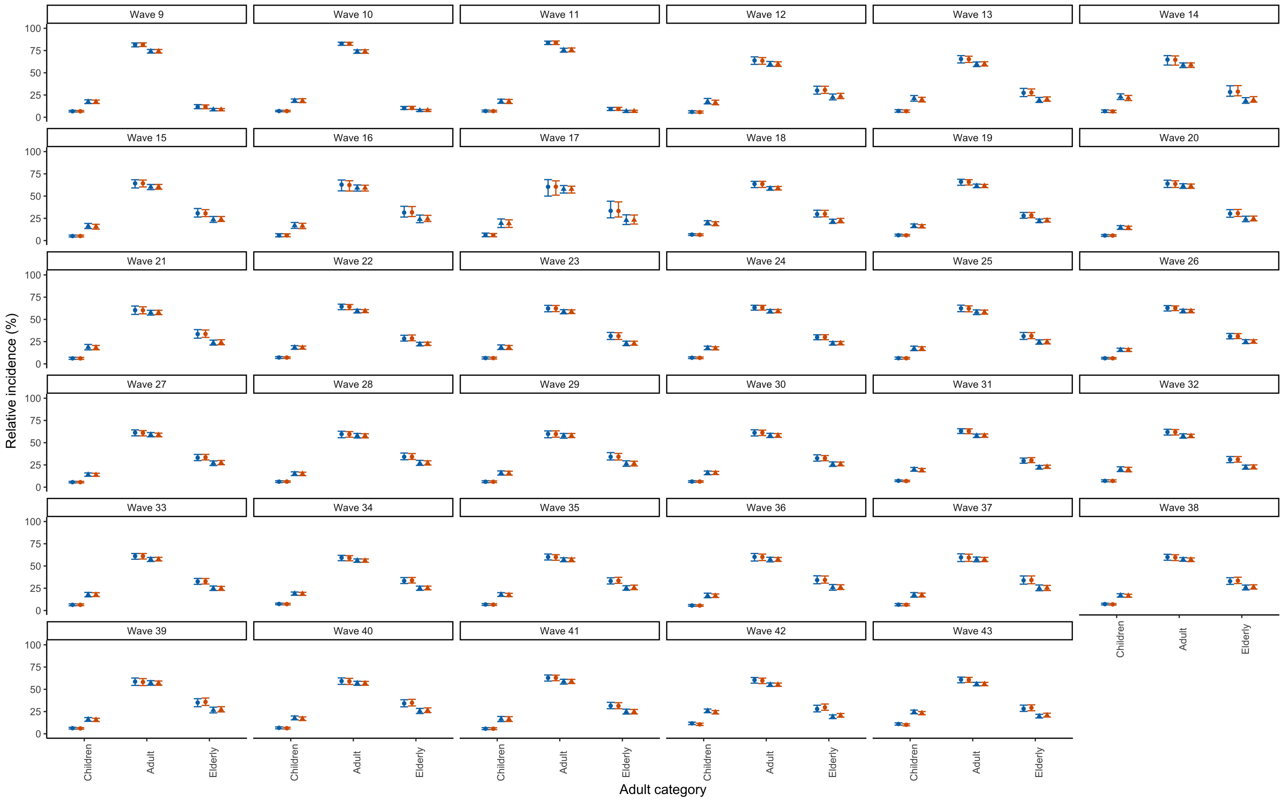

Figure S15: Changes in relative incidence (%) using homogeneous (circle) and heterogeneous (triangle) susceptibility ($a_i = (0.39, 0.83, 0.74)$) and infectivity ($h_j = (0.55, 0.79, 0.99)$) between age categories (Children, Adults, Elderly) per wave [2]; (*Blue*) Without correction (*Orange*) With correction on under-reporting due to fatigue.

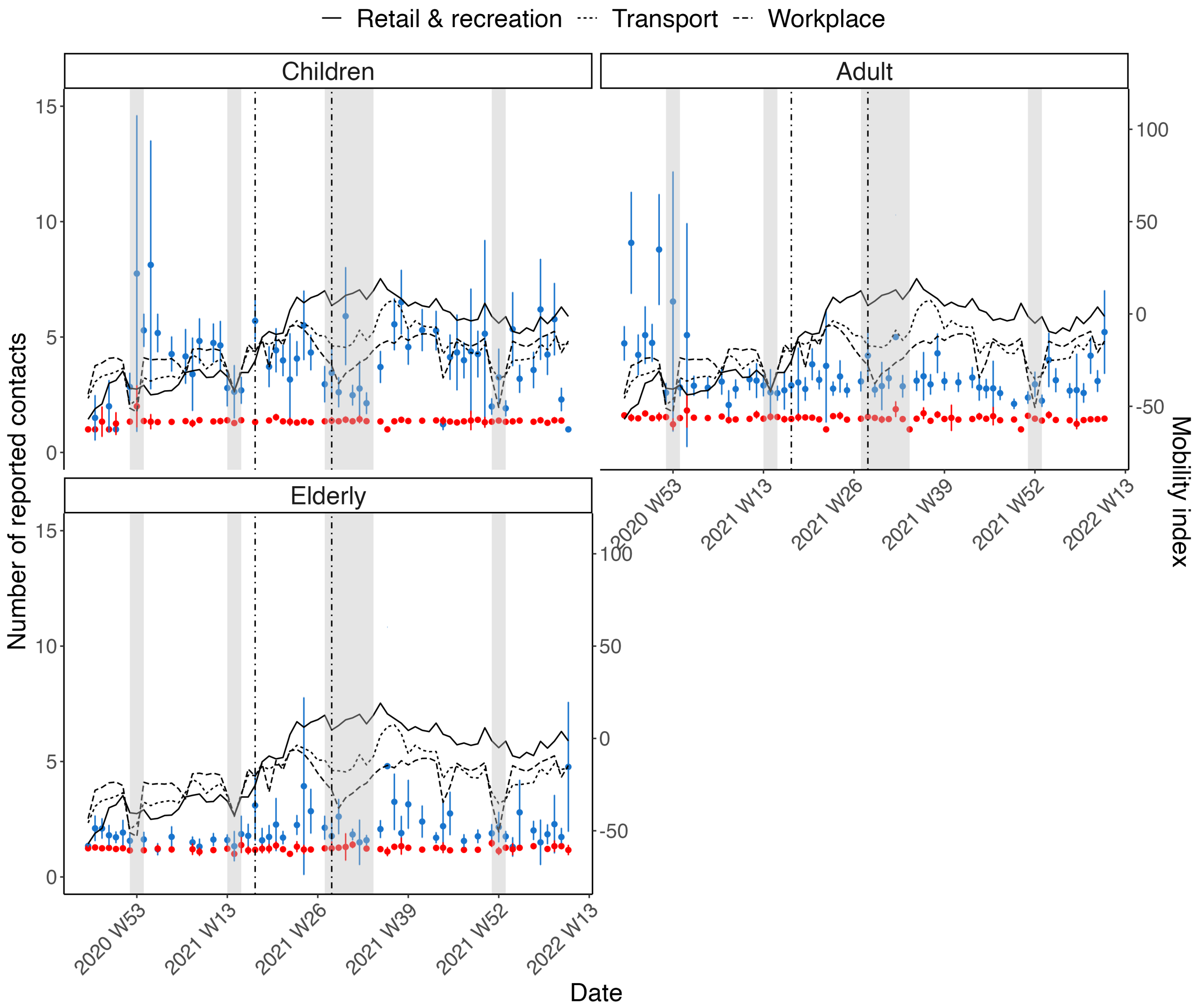

Figure S16: Reported number of contacts of participants in different age groups together with Google Mobility Index. Vertical lines represent the start and the end period of lockdown relaxation in 2021. (Red) household (Black) non-household contacts.

References

[1] Coletti, P., Wambua, J., Gimma, A., Willem, L., Vercruysse, S., Vanhoutte, B., Jarvis, C.I., Van Zandvoort, K., Edmunds, J., Beutels, P., Hens, N.: Comix: comparing mixing patterns in the belgian population during and after lockdown. Scientific Reports **10**(1), 21885 (2020). doi:10.1038/s41598-020-78540-7

[2] Franco, N., Coletti, P., Willem, L., Angeli, L., Lajot, A., Abrams, S., Beutels, P., Faes, C., Hens, N.: Inferring age-specific differences in susceptibility to and infectiousness upon sars-cov-2 infection based on belgian social contact data. PLoS computational biology **18**(3), 1009965 (2022)
