# Supplementary material for: Longitudinal social contact data analysis: insights from 2 years of data collection in Belgium during the COVID-19 pandemic
Source: BMC Public Health. 2023 Jul 6;23:1298. doi: 10.1186/s12889-023-16193-7 (PMC10326964; doi:10.1186/s12889-023-16193-7)
Supplement: Supplementary file 2 — Additional file 2. Connection between GAMLSS for Counts and the Combined Model. This additional file provides the relationship between GAMLSS for counts and the combined model. The derivations depicted the connection between marginal and hierarchical interpretation of Negative Binomial I GAMLSS model. [file 12889_2023_16193_MOESM2_ESM.pdf]

Additional file 2 for: "Longitudinal social contact data analysis: insights from 2 years of data collection in Belgium during the COVID-19 pandemic."

Neilshan Loedy[1*], Pietro Coletti[1], James Wambua[1], Lisa Hermans[1], Lander Willem[2], Christopher I. Jarvis[3], Kerry L.M. Wong[3], W. John Edmunds[3], Alexis Robert[3], Quentin J. Leclerc[3,4,7] Amy Gimma[3], Geert Molenberghs[1,6], Philippe Beutels[2,5], Christel Faes[1], Niel Hens[1,2]

[1]Data Science Institute, I-BioStat, Hasselt University, Hasselt, Belgium.

[2]Centre for Health Economics Research and Modelling Infectious Diseases, Vaccine & Infectious Disease Institute, University of Antwerp, Antwerp, Belgium.

[3]Centre for Mathematical Modelling of Infectious Diseases, Department of Infectious Disease Epidemiology, Faculty of Epidemiology Population Health, London School of Hygiene Tropical Medicine, London, United Kingdom.

[4]Department of Infectious Disease Epidemiology, Faculty of Epidemiology and Public Health, London School of Hygiene Tropical Medicine, London, United Kingdom. [5]School of Public Health and Community Medicine, The University of New South Wales, Sydney, Australia.

[6]L-BioStat, Department of Public Health and Primary Care, Faculty of Medicine, KU Leuven, Leuven, Belgium.

[7]Epidemiology and modelling of bacterial escape to antimicrobials, Institut Pasteur, Paris, France. * Corresponding author: neilshan.loedy@uhasselt.be

2. Additional file 2 : Connection between GAMLSS for Counts and the Combined Model

This additional file provides the relationship between GAMLSS for counts and the combined model. The derivations depicted the connection between marginal and hierarchical interpretation of Negative Binomial I GAMLSS model.

1 Introduction

A connection is established between a particular member of the GAMLSS family for count data, as proposed by Rigby and Stasinopoulos (2010) and the combined model for count data as proposed by Molenberghs, Verbeke, and Demétrio (2007) and studied further in Molenberghs *et al.* (2010). The GAMLSS under investigation is based on the Negative Binomial Type I parameterization of Rigby and Stasinopoulos (2010).

Upon re-parameterization, the models are identical, apart from the choice to place normal random-effects on the variance parameter, in addition to the mean parameter, in the GAMLSS-NBI case.

2 The Combined Model

Molenberghs, Verbeke, and Demétrio (2007) and Molenberghs *et al.* (2010) use the following parameterization for longitudinal (or otherwise hierarchical) count data.

$$\begin{aligned} Y_{ij} &\sim \text{Poi}(\theta_{ij}\kappa_{ij}), & (1)\\ \kappa_{ij} &= \exp\left(\boldsymbol{x}'_{ij}\boldsymbol{\xi} + \boldsymbol{z}'_{ij}\boldsymbol{b_i}\right), & (2)\\ \boldsymbol{b_i} &\sim N(\boldsymbol{0}, D), & (3)\\ E(\boldsymbol{\theta}_i) = E[(\theta_{i1},\ldots,\theta_{in_i})'] &= \boldsymbol{\vartheta}_i, & (4)\\ \text{Var}(\boldsymbol{\theta}_i) &= \Sigma_i. & (5) \end{aligned}$$

Here, Y_{ij} is the count for subject i at time j or, more generically, for repetition j within independent unit i. Further, the conditional mean, given the random effects is:

$$E(Y_{ij}|\boldsymbol{b_i},\boldsymbol{\xi},\theta_{ij}) = \mu^c_{ij} = \theta_{ij}\kappa_{ij}, \tag{6}$$

where the random variable $\theta_{ij} \sim \mathcal{G}_{ij}(\vartheta_{ij}, \sigma^2_{ij})$, $\kappa_{ij} = g(\boldsymbol{x}'_{ij}\boldsymbol{\xi} + \boldsymbol{z}'_{ij}\boldsymbol{b_i})$, ϑ_{ij} is the mean of θ_{ij} and σ^2_{ij} is the corresponding variance. Finally, $\boldsymbol{b_i} \sim N(\boldsymbol{0}, D)$. Write

$$\eta_{ij} = \boldsymbol{x}'_{ij}\boldsymbol{\xi} + \boldsymbol{z}'_{ij}\boldsymbol{b_i}. \tag{7}$$

The generic distribution $\mathcal{G}$ in the count case is chosen to be gamma, in such a way that its two parameters multiply to 1, i.e., is of the form Gamma(α_j, β_j) with $\alpha_j \cdot \beta_j = 1$. This implies that the mean of θ_{ij} equals 1, so that the mean of Y_{ij} equals κ_{ij}.

These authors partially marginalize the conditional distribution over the gamma random effects, leading to:

$$f(y_{ij}|\boldsymbol{b_i}) = \left(\begin{array}{c} \alpha_j + y_{ij} - 1 \\ \alpha_j - 1 \end{array}\right) \cdot \left(\frac{\beta_j}{1+\kappa_{ij}\beta_j}\right)^{y_{ij}} \cdot \left(\frac{1}{1+\kappa_{ij}\beta_j}\right)^{\alpha_j} \kappa_{ij}^{y_{ij}}, \tag{8}$$

where $\kappa_{ij} = \exp[\boldsymbol{x}'_{ij}\boldsymbol{\xi} + \boldsymbol{z}'_{ij}\boldsymbol{b_i}]$. Their motivation is to have a form that depends on normal random effects only, which allows maximization of the corresponding likelihood using a software tool for non-linear mixed-effects models, such as the SAS procedure NLMIXED or the R function `nlme`.

3 Rigby and Stasinopoulos' Negative Binomial Type I Model

Rigby and Stasinopoulos (2005, 2010) propose a general framework where four model elements are allowed to be governed by both fixed and random effects, referred to as GAMLSS. Generically, these four parameters correspond to a mean function, a variance function, a skewness function, and a kurtosis function. It is important to realize that not all four need to be present.

In particular for non-continuous data (e.g., binary data, counts), some functions are induced by others (cf. the mean-variance relationship in certain exponential family models, and hence in the ensuing generalized linear models). The GAMLSS also allows for smoothing parameters.

In their construction of the negative-binomial version of the model, these authors take the following steps:

- The negative-binomial model is generated, in accordance with Breslow (1984), by placing a gamma distribution on the Poisson parameter.
- The parameterization of the gamma distribution is such that it contains a mean and a variance parameter.
- These mean and variance parameters are in turn parameterized by making use of both fixed effects and random effects. The latter random effects are assumed normally distributed.

Rigby and Stasinopoulos (2010, Section 10.14.1, p. 219) present the Negative-binomial Type I model as follows (Type II also exists but is less relevant for us here), where we have introduced the same index system as in (8):

$$p(y_{ij}|\mu_{ij},\sigma_{ij}) = \frac{\Gamma\left(y_{ij}+\frac{1}{\sigma_{ij}}\right)}{\Gamma\left(\frac{1}{\sigma_{ij}}\right)\Gamma\left(y_{ij}+1\right)}\left(\frac{\sigma_{ij}\mu_{ij}}{1+\sigma_{ij}\mu_{ij}}\right)^{y_{ij}}\left(\frac{1}{1+\sigma_{ij}\mu_{ij}}\right)^{1/\sigma_{ij}}. \tag{9}$$

The corresponding moments are:

$$E(Y_{ij}) = \mu_{ij}, \tag{10}$$
$$V(Y_{ij}) = \mu_{ij}+\sigma_{ij}\mu_{ij}^2. \tag{11}$$

4 Connection Between Both Models

Rewrite (8), by expanding the non-integer combinatorial coefficient using Gamma functions, and by allowing the Gamma parameters to depend on j as well:

$$f(y_{ij}|\boldsymbol{b_i}) = \frac{\Gamma\left(\alpha_{ij}+y_{ij}\right)}{\Gamma\left(\alpha_{ij}\right)\Gamma\left(y_{ij}+1\right)}\cdot\left(\frac{\beta_{ij}}{1+\kappa_{ij}\beta_{ij}}\right)^{y_{ij}}\cdot\left(\frac{1}{1+\kappa_{ij}\beta_{ij}}\right)^{\alpha_{ij}}\kappa_{ij}^{y_{ij}}. \tag{12}$$

Comparing (12) with (9) leads to the identities:

$$\alpha_{ij} = \frac{1}{\beta_{ij}} = \frac{1}{\sigma_{ij}},$$
$$\kappa_{ij} = \mu_{ij}.$$

This allows us to write the moments (10)–(11) in both formulations:

$$E(Y_{ij}) = \mu_{ij}=\kappa_{ij}, \tag{13}$$
$$V(Y_{ij}) = \mu_{ij}+\sigma_{ij}\mu_{ij}^2=\kappa_{ij}+\beta_{ij}\kappa_{ij}^2. \tag{14}$$

It is very important that the (partially conditional) mean function depends on $\mu_{ij}\equiv\kappa_{ij}$ only. Indeed, even when both μ_{ij} and σ_{ij} depend on (normally distributed) random effects, the mean function depends only on the random effects in μ_{ij}. This simplifies the full marginalization of the mean, as we will see next.

5 Full Marginalization of the Mean

Expression (13) is the mean function marginalized over the gamma distribution, but with normally distributed random effects present in the linear predictor of the form (7) as in the combined model, or of the form

$$g_1(\boldsymbol{\mu}_i) = X_{i1}\boldsymbol{\xi} + \sum_{k=1}^{K_1} Z_{ik1}\boldsymbol{\gamma}_{ik1}, \tag{15}$$

as in a GAMLSS, with a similar function for the σ_{ij} parameter. Note that the random-effects term on the right hand side of (15) could be written as a single term, by stacking all random effects $\boldsymbol{\gamma}_{ik1}$ and using the Z_{ik1} as the diagonal blocks in a block-diagonal design matrix.

To fully marginalize μ_{ij}, we can make use of Eqn. (35) in Molenberghs *et al.* (2013):

$$\begin{aligned} E(Y_{ij}) &= \int_\theta \int_b \theta_{ij} e^{\boldsymbol{x}'_{ij}\boldsymbol{\xi} + \boldsymbol{z}'_{ij}\boldsymbol{b}_i} \varphi(\boldsymbol{b}_i) d\boldsymbol{b}_i \\ &= E(\theta_{ij}) e^{\boldsymbol{x}'_{ij}\boldsymbol{\xi} + \frac{1}{2}\boldsymbol{z}'_{ij} D \boldsymbol{z}_{ij}} = e^{\ln E(\theta_{ij}) + \boldsymbol{x}'_{ij}\boldsymbol{\xi} + \frac{1}{2}\boldsymbol{z}'_{ij} D \boldsymbol{z}_{ij}}. \end{aligned} \tag{16}$$

Here, $\varphi(\boldsymbol{b}_i)$ is the density of $\boldsymbol{b}_i \sim N(0, D)$. This expression simplifies due to the parameterization used for the Gamma distribution, i.e., $E(\theta_{ij}) = 1$, and hence:

$$E(Y_{ij}) = e^{\boldsymbol{x}'_{ij}\boldsymbol{\xi} + \frac{1}{2}\boldsymbol{z}'_{ij} D \boldsymbol{z}_{ij}}. \tag{17}$$

When other than the unity constraint $\alpha_{ij} \cdot \beta_{ij} \equiv 1$ is used, the logarithmic term in in (16) must be retained.

If further the random-effects structure is limited to a random intercept with variance d, then:

$$E(Y_{ij}) = e^{\boldsymbol{x}'_{ij}\boldsymbol{\xi} + \frac{1}{2}d}.$$

This implies that all parameters in $\boldsymbol{\xi}$, except the intercept, have a marginal interpretation. More generally, if the random-effects structure in (15) is restricted to intercept(s), regardless of the distribution of such effects, the fixed-effects parameters retain their interpretation.

If covariates are present in the random-effects structure, and these are normally distributed, then (17) still offers a parametric description of the marginal mean function, and the corresponding parametric linear predictor function, which will then depend on both $\boldsymbol{\xi}$ as well as D.

References

Breslow, N. (1984). Extra-Poisson variation in log-linear models. *Applied Statistics*, **33**, 38–44.

Molenberghs, G., Kenward, M.G., Verbeke, G., Efendi, A., and Iddi, S. (2013). On the connections between bridge distributions, marginalized multilevel models, and generalized linear mixed models. *International Journal of Statistics and Probability*, **2**, 1–21.

Molenberghs, G., Verbeke, G., and Demétrio, C. (2007). An extended random-effects approach to modeling repeated, overdispersed count data. *Lifetime Data Analysis*, **13**, 513–531.

Molenberghs, G., Verbeke, G., Demétrio, C.G.B., and Vieira, A. (2010). A family of generalized linear models for repeated measures with normal and conjugate random effects. *Statistical Science*, **25**, 325–347.

Rigby, B. and Stasinopoulos, M. (2005). Generalized additive models for location, scale and shape. *Applied Statistics*, **54**, 507–554.

Rigby, B. and Stasinopoulos, M. (2010). *A Flexible Regression Approach Using GAMLSS in R.* Technical Report.
